# Comparative Digital Gene Expression Analysis of Tissue-Cultured Plantlets of Highly Resistant and Susceptible Banana Cultivars in Response to *Fusarium oxysporum*

**DOI:** 10.3390/ijms19020350

**Published:** 2018-01-24

**Authors:** Yuqing Niu, Bei Hu, Xiaoquan Li, Houbin Chen, Tomáš Takáč, Jozef Šamaj, Chunxiang Xu

**Affiliations:** 1 College of Horticulture, South China Agricultural University, Guangzhou 510642, China; 13424453254@163.com (Y.N.); mococo0619@yeah.net (B.H.); hbchen@scau.edu.cn (H.C.); 2 Institute of Biotechnology, Guangxi Academy of Agricultural Sciences, Nanning 530007, China; suzuxiang@163.com; 3 Centre of the Region Haná for Biotechnological and Agricultural Research, Department of Cell Biology, Faculty of Science, Palacký University, 783 01 Olomouc, Czech Republic; tomas.takac@upol.cz (T.T.); jozef.samaj@upol.cz (J.Š.)

**Keywords:** banana *Fusarium* wilt, DGE, Venn diagram, GO annotation, KEGG pathways, resistance genes

## Abstract

Banana *Fusarium* wilt caused by *Fusarium oxysporum* f. sp. *cubense* (*Foc*) is one of the most destructive soil-borne diseases. In this study, young tissue-cultured plantlets of banana (*Musa* spp. AAA) cultivars differing in *Foc* susceptibility were used to reveal their differential responses to this pathogen using digital gene expression (DGE). Data were evaluated by various bioinformatic tools (Venn diagrams, gene ontology (GO) annotation and Kyoto encyclopedia of genes and genomes (KEGG) pathway analyses) and immunofluorescence labelling method to support the identification of gene candidates determining the resistance of banana against *Foc*. Interestingly, we have identified *MaWRKY50* as an important gene involved in both constitutive and induced resistance. We also identified new genes involved in the resistance of banana to *Foc*, including several other transcription factors (TFs), pathogenesis-related (*PR*) genes and some genes related to the plant cell wall biosynthesis or degradation (e.g., pectinesterases, β-glucosidases, xyloglucan endotransglucosylase/hydrolase and endoglucanase). The resistant banana cultivar shows activation of *PR-3* and *PR-4* genes as well as formation of different constitutive cell barriers to restrict spreading of the pathogen. These data suggest new mechanisms of banana resistance to *Foc*.

## 1. Introduction

Banana (*Musa* spp.) is one of the most important fruit and food crops in the world with annual production of more than 100 Mt [1]. *Fusarium* wilt caused by *Fusarium oxysporum* f. sp. *cubense* (*Foc*) is one of the most destructive diseases substantially reducing the production of banana in the world [2,3]. *Foc* has been classified into four physiological races according to the symptoms of different banana cultivars [4]. *Foc* race 4 (*Foc*4) is the most virulent one and affects many *Musa* spp. AAA Cavendish cultivars [3]. Unfortunately, current methods for the management of this disease are not efficient. Therefore, it is of both biological and agricultural importance to understand the molecular mechanism of banana resistance to *Foc*4.

Banana adopted several strategies to cope with *Foc* [5]. Resistant cultivars can avoid the root colonization by inhibiting germination of *Foc* spores [6]. Cell walls of banana root cells undergo reorganization upon *Foc* attack, including pectin methylesterification and changes in the distribution and in the abundance of arabinogalactan proteins and extensins. These dynamic cell wall changes have significant impact on the resistance of banana against *Foc* [7,8]. In addition, genes involved in hormone (jasmonic acid, salicylic acid and ethylene) signalling, canonical pathogen defense genes and antioxidant defense genes have been proposed as candidate genes of banana resistance against *Foc*4 [9,10,11]. As recently suggested, DNA methylation may also contribute to such resistance [12].

Modern bio-techniques, including high-throughput RNA sequencing, are widely employed to study molecular mechanisms of plant growth and development [13], and plant stress defense [14,15]. So far, only a very limited number of studies attempted to reveal the molecular mechanism of banana response to *Foc* using these techniques. For example, general responses of Cavendish banana to *Foc*4 [16] and *Foc*1 were investigated [17] previously. However, a comparison of resistant and susceptible cultivars provides more efficient way to determine the resistance mechanism. Li and coauthors [18] selected a moderately resistant cultivar “Nongke No. 1” and highly susceptible “Brazilian” (in the stage of approximately 30 cm in height) for comparative gene expression analysis focused on metabolic pathways related to the plant immunity. In a similar study, 8-week-old seedlings of highly resistant cultivar “Yueyoukang 1” and highly susceptible “Brazilian” were used by Bai et al. [19] profiting from the release of banana draft genome sequence [20]. They evaluated differential gene expressions in both cultivars after five consecutive infection stages focusing on plant-pathogen interaction and plant hormone signal transduction pathways employing Kyoto encyclopedia of genes and genomes (KEGG) analysis. Both above-mentioned studies highlighted important roles of pathogenesis-related proteins (PRs), signalling, cell wall lignification, hypersensitive response and senesce in the banana resistance against *Foc*4. Nevertheless, detailed information about differences in the gene expression, gene ontology (GO) annotation items and KEGG pathways between resistant and susceptible cultivars was not reported yet.

In this study, tissue-cultured banana plantlets of a highly resistant (“Yueyoukang 1”, *Musa* spp. AAA Cavendish, abbreviated as YK in this study) and susceptible (“Baxijiao”, *Musa* spp. AAA Cavendish, abbreviated as BX in this study) cultivars were subjected to digital gene expression (DGE) analysis before and after *Foc*4 infection. It is known that micropropagated bananas are more susceptible to *Fusarium* wilt than plants grown from conventional material [21]. For this reason, a comparative DGE analysis, exploiting the above mentioned bioinformatic tools, might be more efficient to determine banana resistance mechanisms against *Foc*4. Thus, our approach provides new genes, GO annotation items and KEGG pathways possibly involved in the resistance or susceptibility of banana to *Foc*4. In additional, immunofluorescence labelling method was employed to investigate the changes in the spatio-temporal distribution of xyloglucan in the *Foc*-infected bananas. Most importantly, we have found that enhanced expression of specific transcription factors, receptor-like kinases and genes involved in the cell wall metabolism play a substantial role in the banana resistance to *Foc*.

## 2. Results

### 2.1. Histological Observation of Fusarium Spreading in Root Tissues of Banana Cultivars

**Figure 1:**
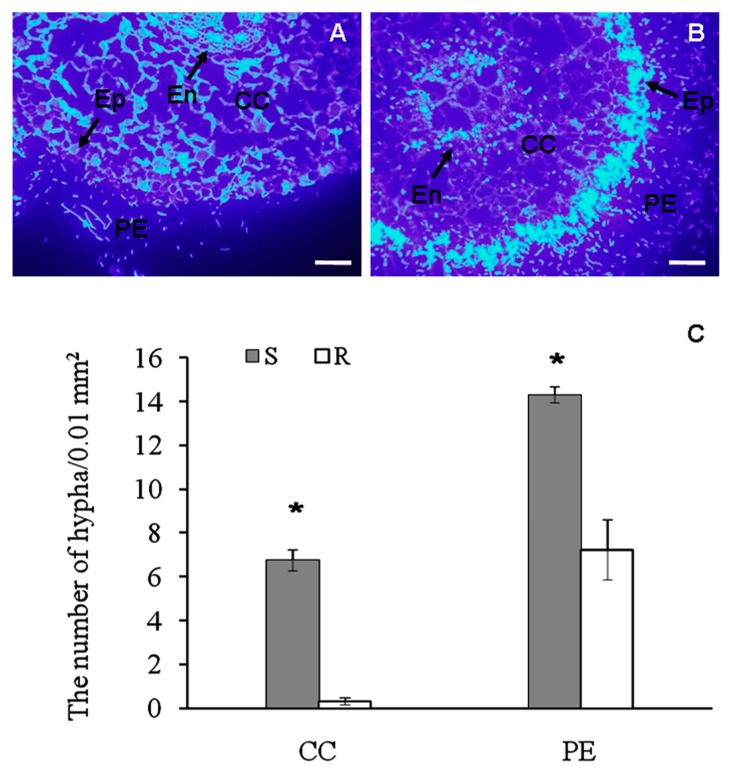
The tissue-specific pathogen spreading in roots of infected banana (*Musa* spp. AAA) seedlings. (**A**,**B**) Visualization of *Fusarium oxysporum* f. sp. *cubense* hyphae by calcofluor white stain in the resistant cultivar “Yueyoukang 1” (**A**) and susceptible “Baxijiao” (**B**) 48 h after infection; (**C**) Quantification of hyphae in A and B. Bars represent 100 μm. CC, cortical cells; Ep, epidermis; En, endodermis; PE, periphery of epidermis; R, resistant cultivar “Yueyoukang 1”; S, susceptible cultivar “Baxijiao”. Quantitative data represent an average of three biological replicates. Comparison of groups was conducted using a paired *t*-test of variance. Values marked with * were considered significant at *p* < 0.05.

Using calcofluor white staining, we attempted to evaluate spreading of *Fusarium* in the inoculated roots of banana seedlings. We observed high density of hyphae at the periphery of epidermis in the resistant cultivar, while it was much less abundant in the endodermis and cortical cells (Figure 1A,C). In contrast, an intense pathogen-derived fluorescent signal was observed in the endodermis and cortical cells of the susceptible cultivar (Figure 1B,C). These results illustrate the resistance of the “Yueyoukang 1” and the susceptibility of “Baxijiao” banana cultivar to *Foc*4 by spatial restriction of the pathogen.

### 2.2. The Quality of Digital Gene Expression (DGE) Data

To identify genes important for banana resistance against *Foc*4, in total eight cDNA libraries were constructed consisting from two biological replications of 4 samples including controls (BXCK, YKCK) and inoculated plantlets (BXT and YKT), and subjected to sequencing in Illumina HiSeq 2000 platform.

As shown in Table S1, clean reads of all tested eight cDNA libraries were more than 10 M and the ratio of clean data to raw data was more than 97% for each library. The percentage of these clean reads that could be mapped to the reference database ranged from 87.59% to 94.29% in all eight libraries. The percentage of unique mapped reads to total clean reads was between 64.63% and 75.79% while it was 17.61–25.83% for multiple mapped reads. The GC content of each sample was more than 50%. The percentage of Q20 and Q30 was more than 98% and 95%, respectively. This evaluation indicated the high quality of DGE data and it could be used for further analysis.

### 2.3. Differential Expression of Genes in the Roots of Banana Cultivars Resistant or Susceptible to Foc4

In our study, we compared gene expression patterns between the cultivars before and after infection. Furthermore, we compared gene expression changes in the response to infection in both cultivars individually. Thus, we evaluated together four different comparison pairs (Table S2). Log2 (Fold Change) > 1 and *p* value < 0.05 were used as the threshold to select significantly differentially expressed genes (DEGs). In total, 955 DEGs were obtained from these four pairs of comparison. They comprised of 575 up-regulated and 380 down-regulated genes. The list of all DEGs including quantification details is presented as Tables S3–S6. Only 32 DEGs (18 up-regulated and 14 down-regulated) were found in the resistant cultivar after pathogen attack compared to the control. On the other hand, 193 DEGs were found in the susceptible cultivar after infection, 54 being up-regulated and 139 down-regulated. After pathogen infection, we found 228 genes with different expression levels between the two cultivars, out of which 159 DEGs showed higher expression levels in the resistant cultivar as compared with the susceptible one (Table S6).

We used a Venn diagram to evaluate genes up-regulated only in the resistant cultivar after infection (as compared to the control) or in the comparison of this cultivar to the susceptible one. As shown in Figure S1A, 12 identified genes fulfilled at least one of these criteria (Table 1), thus they represented genes determining the resistance of banana to *Foc*4. Out of them, four genes (UDP-glycosyltransferase 73C3-like, LOB domain-containing protein 41-like, transcription factor JUNGBRUNNEN 1-like, probable LRR receptor-like serine/threonine-protein kinase At1g74360) were showed higher expression levels in the resistant cultivar compared to the susceptible one after pathogen infection. The expression level of a phenylalanine ammonia-lyase-like gene in the resistant cultivar was significantly higher than that in the susceptible one before pathogen infection while probable WRKY transcription factor (TF) 50 was showed higher expression levels in the resistant cultivar both before and after pathoen infection. Another six genes (luminal-binding protein 2, α-carbonic anhydrase 7-like, curcumin synthase 3-like, proline-rich receptor-like protein kinase PERK3, uncharacterized LOC103972968 and uncharacterized LOC103984595) were transcriptionally induced exclusively in response to the pathogen in the resistant cultivar (Table 1). Forty eight genes were up-regulated only in the susceptible cultivar. There were 286 and 119 DEGs showing higher expression levels in the resistant cultivar in comparison to the susceptible, either before or after the infection, respectively. Together, 33 genes showed higher expression levels in the resistant cultivar when compared to the susceptible one, both before and after pathogen infection. These included some well-known defense related genes, such as PR-4-like, chitinase 1-like and cytochrome P450. They also included some genes related to plant cell wall, such as probable xyloglucan endotransglucosylase/hydrolase protein 23 and β-glucosidase 32-like (Table 2). The detailed functional classification of DEGs revealed by this study is elaborated in the next section.

### 2.4. Gene Ontology (GO) Annotation Items Involved in the Resistance of Banana to Foc4

In order to identify protein functions associated with the resistance of banana against *Foc*4, we performed a GO annotation of DEGs. Only limited numbers of genes were assigned by GO annotations. Five genes (out of 32) differentially regulated after pathogen infection in the resistant cultivar were annotated by 17 GO items. We obtained 99 GO annotations assigned to 33 DEGs (from 193 in total) found after pathogen infection in the susceptible cultivar. Considering DEGs found between the two cultivars before pathogen infection (502 in total), we detected 223 GO annotations for 88 of them. After the infection, 122 GO categories were assigned to 44 genes (from 228 DEGs in total) which were differentially expressed between the two cultivars (Table S7, Figures S2–S5).

Similar to that for DEGs, a Venn diagram was constructed for GO annotation items, classified according to biological process (BP), molecular function (MF) and cellular compartment (CC), for all four pairs of comparison. GO categories common for resistant and susceptible cultivars after pathogen attack represent a general response of banana to *Foc*. They included four CC items (chloroplast, cytoplasmic membrane-bounded vesicle, cytoplasm and plasma membrane) and four MF items (DNA binding, zinc ion binding, chromatin binding and calmodulin binding), but no such items were assigned for BP. Together 26, 6 and 13 GO categories, assigned according to BP (Figure S6A), CC (Figure S6B) and MF (Figure S6C) respectively, appeared exclusively in DEGs found after the infection of the sensitive cultivar (BXCK-BXT). The detailed information of these GO categories is provided in Table S8. Most abundant annotations are related to anaerobic respiration, ammonia assimilation cycle, nitrate assimilation, “de novo” GDP-l-fucose biosynthetic process, glutamate metabolic process, glutamate decarboxylase activity, nonphotochemical quenching, development and photosynthesis.

However, for DEGs found exclusively after the infection of the resistant cultivar (YKCK-YKT), only two GO categories were found according to BP; cellular zinc ion homeostasis and one-carbon metabolic process) and two GO items were found according to MF (carbonate dehydratase activity and calcium-transporting ATPase activity). Five other GO categories belonging to BP (RNA splicing, calcium ion transport, pollen development, regulation of transcription, DNA-templated, response to nematode) were present in the comparison of YKCK-YKT and BXCK-YKCK or BXT-YKT or both (Figure S6).

Furthermore, 37 GO annotation items were found exclusively between the two cultivars both before and after pathogen infection. They include 18, 11 and 8 GO annotation items assigned to BP, CC and MF, respectively (Figure S6, detailed information is listed in Table 3). Most abundant annotations include plant cell wall biosynthesis and degradation and responses to stresses. Among these 37 GO annotation items, those related to plant cell wall biosynthesis and degradation belonged the top 10 abundant annotations, such as cellular polysaccharide metabolic process, chitinase activity, pectinesterase activity and xyloglucan:xyloglucosyl transferase activity.

Table 4 shows 10 significantly accumulated GO categories and 18 genes included in these GO items, considering corrected *p*-value ≤ 0.05 as threshold. Pathogen infection caused significant accumulation in five (assigned to two genes) and four GO categories (assigned to eight genes) in the resistant and susceptible cultivars, respectively. In the resistant cultivar, the significantly accumulated GO categories include cellular zinc ion homeostasis, RNA splicing, one-carbon metabolic process, carbonate dehydratase activity and calcium-transporting ATPase activity. In the susceptible cultivar, significantly accumulated GO categories were anaerobic respiration, iron ion binding, heme binding and electron carrier activity. After pathogen infection, GO annotation named DNA binding was significantly accumulated in the susceptible but not in the resistant cultivar. There was only one GO annotation item which included nine genes. However, there were no significantly accumulated GO items between two cultivars before pathogen infection, although the number of GO categories was the highest.

**Figure 2:**
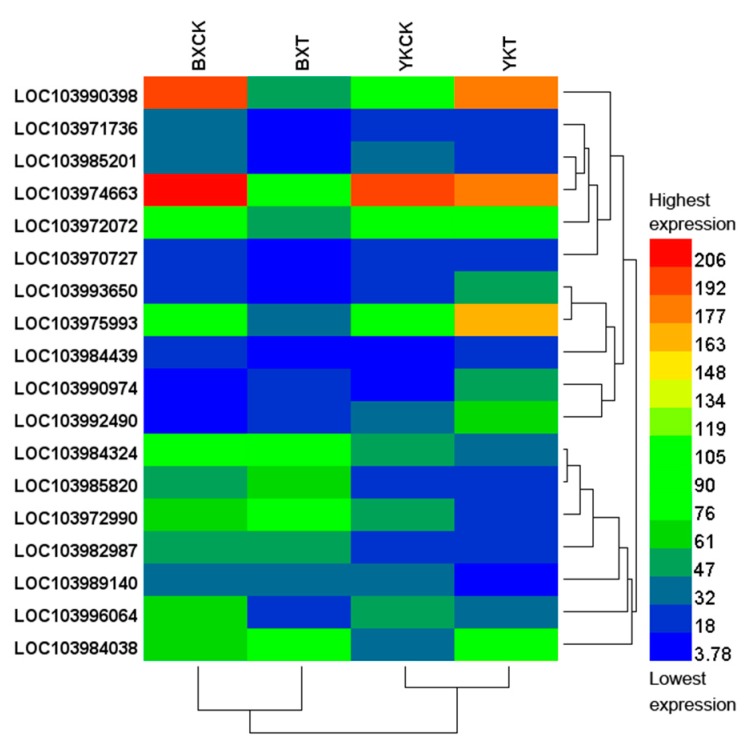
Evaluation of the 18 genes classified in the top 10 GO annotations using heat map in terms of their RPKM values. Red represents the highest expression level while blue represents the lowest one. BX: “Baxijiao”, highly susceptible to *Fusarium oxysporum* f. sp. *cubense* race 4; YK: “Yueyoukang 1”, highly resistant to *Fusarium oxysporum* f. sp. *cubense* race 4. CK: control; T: treatment with pathogen. GO: gene ontology; RPKM: reads per kilobase of transcript per million mapped reads.

Next, we attempted to evaluate 18 genes classified in the top ten GO annotations (Table 4) using a heat map in terms of their reads per kilobase of transcript per million mapped reads (RPKM) values. As shown in Figure 2, these 18 genes could be grouped into the following gene clusters: (1) four genes (LOC10394439, LOC103993650, LOC103990398 and LOC103975993) accumulated in the resistant cultivar after pathogen infection, but it was opposite in the susceptible one; (2) Genes (LOC103992490, LOC103984038 and LOC103990974) with increased expression after pathogen infection in both cultivars but more intensive increase was observed in the resistant cultivar; (3) Genes with increased expression in the sensitive cultivar, but decreased or stable in the resistant cultivar (LOC103984324, LOC103972990 and LOC103985820); (4) Genes with decreased expression in the susceptible cultivar but less or nearly no decrease in the resistant one (LOC103971736, LOC103970727 and LOC103972072); (5) Genes with decreased expression in both cultivars but more intensive decrease was observed in the susceptible cultivar (LOC103985201, LOC103996064 and LOC103974663); (6) Genes which expression decreased significantly in the resistant cultivar, but with no change in the susceptible one (LOC103989140); (7) Genes not responding to the pathogen but showing significantly higher expression in the susceptible cultivar than in the resistant one both before and after pathogen infection (LOC103982987).

### 2.5. Kyoto Encyclopedia of Genes and Genomes (KEGG) Analysis

DEGs found between the two cultivars before and after pathogen infection were classified into 67 (corresponding to 113 genes) and 47 (corresponding to 45 genes) KEGG pathways, respectively. On the other hand, 14 (corresponding to 8 genes) and 51 (corresponding to 59 genes) KEGG pathways were found after the pathogen infection in the resistant and susceptible cultivar, respectively (Table S7, Figures S7–S10). Together we detected 86 different KEGG pathways and ten of them appeared in all four pairs of comparison. Thirteen KEGG pathways were found simultaneously in DEGs detected at least in three pairs of comparison including BXCK-BXT, YKCK-YKT and BXT-YKT or/and BXCK-YKCK. This suggests that they are generally involved in the responses of banana to *Foc*4. Six pathways (fructose and mannose metabolism, sphingolipid metabolism, butanoate metabolism, porphyrin and chlorophyll metabolism, carotenoid biosynthesis and ribosome) are exclusively present after the pathogen infection of the sensitive cultivar. On the other hand, there was only one pathway (protein export) exclusively present after the pathogen infection of the resistant cultivar. Numbers of accumulated KEGG pathways exclusively present in BXCK-YKCK and BXT-YKT comparisons were 15 and 9, respectively (Figure S11). Ten KEGG pathways, including steroid biosynthesis, carbon fixation in photosynthetic organisms and several pathways related to amino acid metabolism, were present in both of BXT-YKT and BXCK-YKCK comparisons (Figure S11 and Table 5).

When considering corrected *p* value ≤ 0.05 as the threshold, 21 KEGG pathways showed significant enrichment (Table 6 and Table S9). Ten significantly enriched items were found in DEGs after pathogen infection in the susceptible cultivar but only one in the resistant one (circadian rhythm—plant). Among DEGs found between the two cultivars before pathogen infection, ten significantly enriched KEGG pathways were identified; however, there was no significantly enriched KEGG pathway after the infection.

### 2.6. Changes in Thespatio-Temporal Distribution of Xyloglucan in Foc-Infected Banana

**Figure 3:**
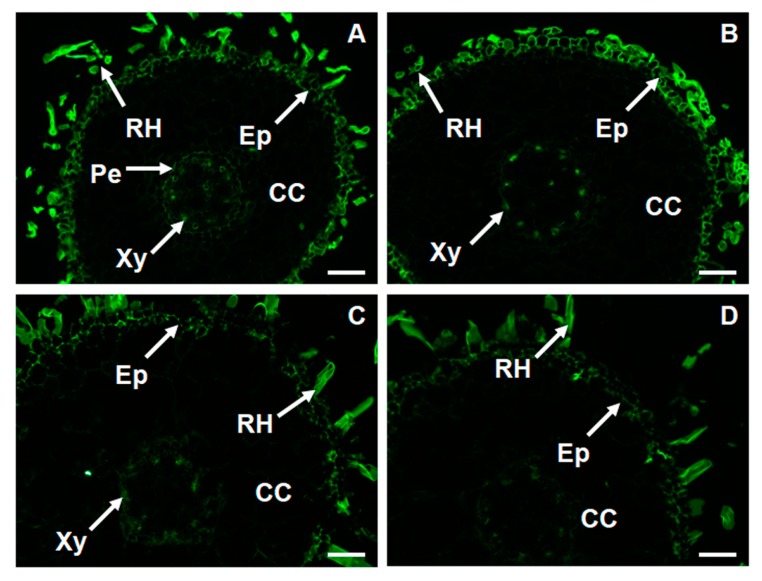
Immunolocalization of xyloglucan in banana (*Musa* spp. AAA) root cross sections by LM15 antibody. (**A**) Resistant cultivar before infection with *Foc*4; (**B**) Resistant cultivar after infection with *Foc*4; (**C**) Susceptible cultivar before infection with *Foc*4; (**D**) Susceptible cultivar after infection with *Foc*4; Bars represent 100 μm. CC, cortical cells; Ep, epidermis; *Foc*4, *Fusarium oxysporum* f. sp. *cubense*; Pe, pericycle; RH, root hairs; Xy, xylem.

As mentioned above, we have found several genes and GO annotation items related to plant cell wall hemi-cellulose perhaps involved in banana resistance to *Foc*4. In order to validate these results, we conducted immunofluorescent histological observations of xyloglucan using LM15 monoclonal antibody in root sections of the two banana cultivars. We observed strong fluorescent signal in root hairs and epidermis while slightly weaker signal was detected in xylem and pericycle. Remarkably, fluorescent signal of lower intensity was observed in the susceptible cultivar. Pathogen infection caused obvious increase in the signal intensity in the epidermis and in the xylem of the resistant cultivar. Unlike in the resistant cultivar, *Foc*4 infection did not enhance the fluorescence signal in the susceptible cultivar (Figure 3).

### 2.7. Quantitative Real-Time PCR (qRT-PCR) Validation of DGE Data

To confirm the results of the Illumina Solexa sequencing, eight unigenes for quantitative real-time PCR (qRT-PCR) assays were selected from genes included in the significantly enriched GO annotations or KEGG pathways. These were: LOC103987502 (chalcone synthase 2-like), LOC103972276 (tropinone reductase homolog At1g07440-like), LOC103995575 (pathogenesis-related protein 1-like), LOC103978702 (chitinase 1-like), LOC103992490 (NAC domain-containing protein 68-like), LOC103990974 (TF JUNGBRUNNEN 1-like), LOC103993974 (probable 2-aminoethanethiol dioxygenase), LOC103974663 (trans-cinnamate 4-monooxygenase-like). As shown in Figure S12, six of them were consistent with the DGE analyses. These results approved Illumina Solexa sequencing data.

## 3. Discussion

We carried out a DGE analysis to screen which genes are specifically responsible for *Fusarium* resistance at the young seedling stage for better understanding of the mechanism underlying the resistance of banana to *Foc*4.

The studied two cultivars significantly differ in the resistance to *Foc*4, as shown by the high abundance of the pathogen hyphae in the inner root tissues of the susceptible cultivar. Resistant cultivar likely developed effective mechanism to minimize the spread of the pathogen, which remained predominantly at the root periphery. As proposed by our DGE analysis, resistant cultivar is equipped with unique set of genes involved in various functions to minimize colonization by the pathogen. These genes are discussed below.

We present a general view on differences in the gene expression patterns between two banana cultivars with *Foc* resistance or susceptibility using various bioinformatic tools. Enriching results reported by Bai et al. [19], more genes were found to be differently expressed in the susceptible cultivar as compared to the resistant one in the present study. This might be caused by higher susceptibility of tissue-cultured young banana plantlets [21] used in our study in comparison to older plants used by Bai et al. [19], and by in vitro conditions.

### 3.1. Receptor Like Kinases

Two genes acting as receptor-like kinases were identified as determinants of banana resistance against *Foc*4. These kinases are involved in the sensing and perception of the pathogen derived signal [22], therefore they might be important for the banana resistance against *Foc*4. One of them, *probable LRR receptor-like serine/threonine-protein kinase At1g74360* was previously reported to be required for immunity against parasitic nematodes in *Arabidopsis* [23]. Proline-rich receptor-like protein kinase PERK3 belongs to the receptor kinases which share a putative extracellular domain related to cell wall proteins. They are suggested to sense changes induced by pathogens in the cell wall [24].

### 3.2. Trancription Factors

Plant TFs are involved in the regulation of defense-related gene expression against different types of microbial pathogens. For example, Jin et al. [25] suggested that, ethylene-responsive factor (ERF) *CaPTI1* gene regulates defense response of pepper (*Capsicum annuum* L.) to *Phytophthoracapsici*. Liu et al. [26] found that *SpMYB* TF expression was significantly induced after *Arabidopsis* infection with *Fusarium oxysporum* or *Botrytis cinerea*. Furthermore, *SpMYB* overexpressing transgenic tobacco plants are more resistant to *F. oxysporum* and *B. cinerea*as compared to the wild-type plants, suggesting that *SpMYB* positively regulates plant disease resistance. In banana, up-regulated TFs including *WRKY6*, *WRKY26*, *WRKY31*, *WRKY33*, *WRKY65* and *WRKY72*, ethylene responsive TFs, such as ethylene insensitive 3 (*EIN3*) and ethylene insensitive 3-like 1 (*EIL1*) were found in *Foc*-resistant cultivar “Nongke 1” [18]. Bai et al. [19] also reported that three TFs including *WRKY22*, *WRKY33* and *DREB* showed different expression patterns between *Foc*-resistant and susceptible banana cultivars. The *NAC* (*NAM, ATAF1/2*, *CUC2*) gene family encodes a large family of plant-specific TFs that play diverse roles in plant development and stress regulation. Based on our study, NAC domain-containing protein 68-like determines the resistance of banana to *Foc*. On the other hand, the expression of another member of this TF family, NAC domain-containing protein 21/22-like, positively correlated with the increased susceptibility to *Foc*. The involvement of *NAC21/22* in the susceptibility of plants to diseases was also reported in wheat (*Triticum aestivum*) [27]. Chen et al. [28] revealed that over-expression of *SmNAC* decreases resistance to bacterial wilt. These data suggest that plant NAC could play an important function in plant-pathogen interaction.

*WRKY* TFs are established regulators of defense genes. Products of these genes might be phosphorylated by mitogen activated protein kinases [29]. In the present study, probable *MaWRKY50* was up-regulated by the pathogen exclusively in the resistant cultivar. Furthermore, this TF was more expressed in the resistant cultivar as compared to the susceptible one, both before and after pathogen infection. These results suggested that *MaWRKY50* is an important TF determining both basal and induced resistance of banana to *Foc*4. *WRKY50* is well-known TF participating in the salicylic acid signaling pathway in *Arabidopsis* [30]. Other promising TF candidates supporting the banana resistance against *Fusarium* might be JUNGBRUNNEN1 (JUB1) and LATERAL ORGAN BOUNDARIES (LOB) DOMAIN (LBD)-containing protein 41-like. In contrast to banana, JUB1 and LOB 20 have been shown rather to suppress the defense responses of *Arabidopsis* against *Pseudomonas syringae* and *Fusarium oxysporum* [31,32].

### 3.3. Canonical Defense Related and Cell Wall Associated Genes

Several canonical defense-related genes exert higher expression in the resistant cultivar before and after the infection (Table 2). For example, cytochrome P450 is the member of jasmonic acid pathway, a well-known signalling pathway associated with plant defense [33]. Many biochemical conversions in plant steroid hormone biosynthesis are catalyzed by cytochrome P450 enzymes (*CYPs* or *P450s*). Recently, Yang et al. [34] found that heterologous expression of wild eggplant (*Solanummelongena*) cytochrome P450 gene, *StoCYP77A2*, in tobacco confers enhanced disease resistance against *Verticillium dahliae*.

PR proteins play a very important role in plant innate immunity. PR-1 is one of the most abundant from this family of proteins. In the present study, PR-1 like (LOC103977651 and LOC103977653) was up-regulated by the pathogen exclusively in the susceptible cultivar. Another *PR-1* member, LOC103975649, was down-regulated by the pathogen in the resistant cultivar. Differently, some other *PRs*, such as *PR-3* and *PR-4*, belonging to chitinases, showed significantly higher expression levels in the resistant cultivar than in the susceptible one, both before and after pathogen infection. Chitinases are in the first line of plant defense response. They can disrupt fungal cell walls and produce chitin oligomers eliciting the plant defense signalling pathways [35]. They have been shown to be key players in the response to *Foc* infection in Cavendish banana [18]. The fact that not all *PR* genes (see *PR-1*) are induced in the resistant banana cultivar, might suggest that this cultivar activates different defense cascades including activation of *PR-3* and *PR-4* or accumulation of hemicelluloses to form different constitutive cell barriers.

Significant number of genes is involved in cell wall metabolism, indicating that cell wall, as a mechanical barrier preventing pathogen spreading, may be one of the crucial mechanisms of banana resistance. This is also reflected by enrichment of cell wall related genes after the infection observed by the KEGG pathway analysis. Specific remodeling of cell wall components such as extensins, arabinogalactan proteins and pectins substantially enhances the resistance of banana to *Fusarium* [7,8]. We identified enzymes metabolizing xylan and xyloglucans, which are important cell wall structural constituents [36]. These enzymes contribute to the banana resistance against *Foc*4. Immunofluorescence observation of xyloglucan validated these results and showed their profound accumulation in epidermis of the resistant cultivar. This is also in agreement with the accumulation of the pathogen in the epidermal periphery (Figure 3). Consistent outputs were obtained by comparative GO annotation. Several cell wall biosynthesis or degradation-related GO annotations were found to be preferentially enriched in the resistant cultivar (e.g., cellular glucan metabolic process, cellular polysaccharide metabolic process, pectinesterase activity, xyloglucan:xyloglucosyl transferase activity and transferase activity, transferring glycosyl groups). Our previous work revealed that the susceptible banana cultivar showed higher pectinesterase activities both before and after *Foc*4 infection, but pathogen infection lowered activity of this enzyme in both cultivars [7]. This was consistent with the expression levels of LOC103975191, the only gene assigned to GO: 0030599 (pectinesterase activity).

### 3.4. Secondary Metabolism

Our data also indicate that similarly to other species [37], secondary metabolites play very important roles in plant pathogen defense. Enzymes involved in phenyl-propanoid secondary metabolites or curcumin have been up-regulated preferentially in the resistant banana cultivar after the *Foc*4 infection.

### 3.5. New Genes with Putative Role in Foc4 Resistance

Several genes, which were preferentially up-regulated in the resistant cultivar, were not reported in plant disease resistance so far (e.g., *α-*carbonic anhydrase 7-like, phenylpropanoylacetyl-CoA synthase-like, probable small nuclear ribonucleoprotein G, hexose carrier protein HEX6, protein GOS9-like). They are promising candidates for regulators of banana resistance against *Foc*4. However, the precise mechanism of their function remains to be elucidated in the future.

### 3.6. Comparative GO and KEGG Analysis and Screening of Potential Resistant Genes

We provide here a comprehensive comparative elaboration of KEGG pathways important for resistance or sensitivity of banana against *Foc*. Taking corrected *p* value ≤ 0.05 as the threshold, there was only one KEGG pathway, circadian rhythm—plant, exclusively enriched in inoculated resistant cultivar. Circadian clock is the internal time-keeping machinery which helps plants to anticipate diurnal changes [38]. In the recent years, many lines of evidences, including our study, showed that the circadian rhythm modulate plant immune responses [39,40].

Further, ten KEGG pathways which were enriched between the two cultivars both before and after pathogen attack might be responsible for the basal resistance/susceptibility of banana against *Foc*. Most of these pathways were not reported in previous works [18,19], which might be caused by higher susceptibility of tissue-cultured young banana plantlets [21]. Three of them belong to amino acid metabolism (glycine, serine and threonine metabolism, valine, leucine and isoleucine degradation, lysine degradation). Recently, plant amino acid metabolism was also found to be involved in plant resistance to diseases. For example, Seifi et al. [41] found that infection of wild-type tomato (*Solanum lycopersicum*) leads to a strong transcriptional up-regulation of asparagine synthetase, followed by a severe depletion of asparagine titers. In contrast, resistant *sitiens* tomato plants displayed a strong induction of asparagine throughout the course of infection, and asparagine synthetase played an immune-regulatory role in *Botrytis cinerea*-tomato interaction. Over-expression of cytosolic aspartate amino transferase not only led to changes in the content of aspartate and aspartate-derived amino acids, but also affected defense responses against *B. cinerea* infection in *Arabidopsis thaliana* [42]. Thus, amino acid metabolism likely contributes to the defense strategy against *Foc*4 in banana. Citrate cycle, TCA cycle and Krebs cycleare important aerobic pathways for the final steps of the oxidation of carbohydrates and fatty acids. The DEGs assigned in this KEGG pathway showed higher expression levels in the susceptible cultivar than in the resistant one both before and after infection. In addition, GO annotation analysis also showed that GO annotation items, such as anaerobic respiration, aromatic amino acid family biosynthetic process, cysteine biosynthetic process, and glutamate metabolic process were detected exclusively in the susceptible cultivar (Table S8). These suggest higher demand for energy in the susceptible cultivar to cope with the negative effects of the pathogen.

## 4. Materials and Methods

### 4.1. Materials

Two banana cultivars, *Musa* spp. AAA cv. “Baxijiao” and “Yueyoukang 1” were used in this study. “Yueyoukang 1”, a cultivar from Cavendish selection GCTCV-218, is highly resistant to *Foc*4 (disease incidence in the field is under 5%) while “Baxijiao” is highly susceptible to this pathogen (with disease incidence of 44.4% in the field [43]).

Single-spore cultures of highly virulent *Foc*4 were used in the present study for banana inoculation. The pathogen cultivated on solid potato dextrose agar (PDA) medium (200 g/L patato + 20 g/L dextrose + 13 g/L agar) and kept at 4 °C for two cycles was activated on the fresh PDA medium and cultivated at 28 °C for 3 days in dark before transferring to the liquid potato lactose medium (200 g/L patato + 20 g/L lactose + 13 g/L agar) to produce spores. After 7-day-culture on a shaker rotating at 140 rpm, spore suspension was ready for use.

### 4.2. Inoculation of Banana Cultivars with Pathogen

Preparation of pathogen and the inoculation of two banana cultivars with this *Foc*4 were carried out according to the protocol described in our previous work [7]. In brief, tissue cultured banana seedlings without roots were cultivated two weeks in liquid rooting medium (MS [44] + 0.1 mg/L indole-3-butyric acid + 0.2 mg/L α-naphthaleneacetic acid). One newly emerged root (0.5–1.0 mm in diameter) of each plant was cut off to facilitate the penetration of the pathogen and it was transferred to a new rooting medium containing *Foc*4 at a final concentration of 5 × 10^2^ conidia per mL (inoculation treatment). Control plants were cut-treated likewise, and they were transferred to a rooting medium without fungus.

Roots (devoid of excess liquid media by brief blotting on filter paper) were collected 24 h after treatments. One biological replicate of each individual sample consisted of whole roots from 21 seedlings. RNA extracted from the roots was subjected to DGE and qRT-PCR analysis. Values reported represent the average of three and two biological replicates for qRT-PCR and DGE, respectively. Individual roots were used for histological study.

### 4.3. Immunolabelling of Xyloglucan

Samples were collected 24 h after treatments. Fixation and embedding of samples were carried out as described in Xu et al. [45]. In detail, root tips of about 1 cm in length were fixed in 3.7% (*v*/*v*) formaldehyde in stabilizing buffer MTSB (50 mM piperazine-*N*,*N*′-bis(2-ethanesulfonic acid), 5 mM MgSO_4_·7H_2_O, 5 mM ethyleneglycol bis (2-aminoethylether)-*N*,*N*,*N*′,*N*′-tetraacetic acid (EGTA), pH 6.9) for 1 h at room temperature, dehydrated in a successive ethanol series (30%, 50%, 70%, 90%, and 100%; *v*/*v*) and embedded in Steedman’s wax [46]. Thin sections (8–10 μm) were de-waxed and rehydrated in a successive ethanol series (100%, 90%, 70% and 50%; *v*/*v*), blocked in phosphate-buffered saline (PBS) supplemented with 50 mM glycine and 2% (*w*/*v*) bovine serum albumin (BSA). To detect the spatio-temporal distribution of xyloglucans, tissue sections were labelled with LM15, a major primary monoclonal antibody (diluted 1:20 in PBS containing 1% (*w*/*v*) BSA) recognizing XXXG motif of xyloglucan [47] at 4 °C overnight. After washing in PBS three times, sections were incubated in anti-rat IgG secondary antibody conjugated with FITC diluted 1:20 in PBS containing 1% (*w*/*v*) BSA for 1 h at room temperature. Afterwards, the sections were washed with PBS three times and they were stained with 0.01% of toluidine in PBS for 10 min to quenchtissue auto-fluorescence. Finally, the sections were rinsed with PBS (three times, each for 10 min) and mounted in anti-bleach medium before observation. Sections probed only with secondary antibodies were used as controls. Three biological replicates each consisting of 2 sections were prepared for individual treatment. Fluorescence was examined with an Imager D2 (ZEISS, Oberkochen, Germany). Exposure time was 30 ms.

### 4.4. Observation of Pathogen Diffusion in Root Tissues of Banana

The protocols for fixation and embedding of samples (collected 48 h after treatments) were carried out as described above. To observe the differences in pathogen diffusion between the resistant and susceptible cultivars, de-waxed cross sections of roots were stained with Calcofluor White Stain (Sigma-Aldrich, Saint Louis, MO, USA) for 10 min followed by three times of rinse with PBS buffer. Images were taken by using UV light for excitation of with a Olympus BH-2-FRCA microscope (Olympus, Tokyo, Japan).

The quantification of the hyphae represented an average of three biological replicates (one biological replicate was calculated as an average hypha number counted from 10 banana cell area of 0.01 mm^2^) ± standard deviation. A comparison of groups was conducted using a paired *t*-test of variance.

### 4.5. DGE Analysis

RNA preparation, library preparation for DGE sequencing and data analysis were carried out as described in our former paper [8].

### 4.6. GO and KEGG Enrichment Analysis of Differentially Expressed Genes

The GO enrichment analysis of DEGs was carried out by Blast2Go software [48]. GO terms with corrected *p* value less than 0.05 were considered as significantly enriched by DEGs. Application of Blast2GO algorithm for GO function classification enabled to get three categories of all the sequences in GO, respectively: MF, CC, BP. KEGG is a database resource for understanding high-level functions and utilities of the biological system, from molecular-level information, especially large-scale molecular datasets generated by genome sequencing and other high through-put experimental technologies (http://www.genome.jp/kegg/). We used KOBAS software to test the statistical enrichment of DEGs in KEGG pathways.

### 4.7. qRT-PCR Validation Result

The total RNA was extracted as described previously [8]. RNA was reverse transcribed in a 10 uL reaction system using the PrimeScriptTM RT Master Mix Kit (TaKaRa, Otsu, Japan). Gene-specific primers were designed base on the gene sequences using Primer 3.0 software and the primer sequences are listed in Table S10. The 18S rRNAgene of banana was used as a reference gene. The qRT-PCR was performed using the SYBR Premix Ex Taq Kit (TaKaRa, Otsu, Japan) according to the manufacturer’s protocol. And all qRT-PCR reactions were carried out using Thermal Cycler Dice (TaKaRa, Otsu, Japan). Individual reactions were run with each primer pair with annealing temperatures ranging from 55 to 60 °C. The conditions were as follows: initial holding at 95 °C for 3 min, followed by a two-step program of 95 °C for 10 s and annealing temperature for 30 s for 40 cycles. Each sample was analyzed in three technical replicates. The relative changes in gene expression levels were calculated using the2^−ΔΔ*C*t^ method [49].

### 4.8. Statistical Analysis

The DEGs of the resistant and susceptible banana cultivars before and after infection with *Foc*4 were compared with the method described by Audic et al. [50]. The false discovery rate (FDR) was used to determine the threshold of *p* value in multiple test and analysis. A threshold of FDR < 0.001 was used to judge the significance of gene expression difference. *p* ≤ 0.01, FDR ≤ 0.1, and the absolute value of log2 ratio ≥ 1 were used as threshold to assess the significance of gene expression difference.

## 5. Conclusions

Thank to comparative bioinformatic analyses and exploitation of tissue-cultured plants, we were able to provide a unique set of genes which are important for banana resistance to *Foc*. The results showed that in addition to canonical *PR* genes, increased expression of TFs, receptor-like kinases, cell wall regulatory enzymes as well as metabolic genes play important roles in the resistance of banana to *Foc*. The resistant banana cultivar developed structural barriers for restricting the pathogen spread. The results obtained in the present study will help to better understand banana resistance mechanism against *Foc*. Precise function and the regulation of the most important genes related to the resistance of banana to *Foc*4 should be of main interest for future studies, and for generation of resistant banana germplasm using genetic modification [51].

## Figures and Tables

**Table 1:** Genes up-regulated by *Fusarium oxysporum* f. sp. *cubense* race 4 exclusively in the resistant banana (*Musa* spp. AAA) cultivar and genes showing higher expression levels in the resistant cultivar in comparison to the susceptible one after the infection.

Gene ID	Gene Description
LOC103991268	Probable WRKY transcription factor 50
LOC103971627	Phenylalanine ammonia-lyase-like
LOC104000780	Probable LRR receptor-like serine/threonine-protein kinase At1g74360
LOC103983205	UDP-glycosyltransferase 73C3-like
LOC103980149	LOB domain-containing protein 41-like
LOC103990974	Transcription factor JUNGBRUNNEN 1-like
LOC103978674	Luminal-binding protein 2
LOC103984595	Uncharacterized
LOC103984038	α-carbonic anhydrase 7-like
LOC103968789	Curcumin synthase 3-like
LOC103983427	Proline-rich receptor-like protein kinase PERK3
LOC103972968	Uncharacterized

LOB. lateral organ boundaries; LRR: leucine-rich repeat; UDP: uridine diphosphate.

**Table 2:** Genes with higher expression levels in the resistant banana (*Musa* spp. AAA) cultivar before and after infection with *Fusarium oxysporum* f. sp. *cubense* race 4.

Gene ID	Gene Description
LOC103993913	Uncharacterized
LOC103978702	Chitinase 1-like (*PR-3*)
LOC104000237	Momilactone A synthase-like
LOC103978704	Chitinase 1-like (*PR-3*)
LOC103989437	Protein GOS9-like
LOC103975319	Uncharacterized protein At1g15400-like
LOC103969557	Transmembrane protein 45A-like
LOC103970573	β-glucosidase 32-like
LOC103992490	NAC domain-containing protein 68-like
LOC103977235	Phenylpropanoylacetyl-CoA synthase-like
LOC103989217	Uncharacterized
LOC103989972	PR-4-like
LOC103992834	Putative calcium-transporting ATPase 13, plasma membrane-type
LOC103988092	Scarecrow-like protein 23
LOC103980421	Uncharacterized
LOC103993506	Uncharacterized
LOC103974823	α-carbonic anhydrase 7-like
LOC103987744	Cytochrome P450 CYP72A219-like
LOC103979138	Cytochrome P450 71A1-like
LOC104000899	Uncharacterized
LOC104000297	Leucine-rich repeat receptor protein kinase EXS
LOC103974226	Uncharacterized
LOC103977900	Uncharacterized
LOC103989489	Miraculin-like
LOC103983075	Uncharacterized
LOC103976880	Probable xyloglucan endotransglucosylase/hydrolase protein 23
LOC103979054	Calcium-binding protein PBP1-like
LOC103974727	Probable small nuclear ribonucleoprotein G
LOC103992513	Probable WRKY transcription factor 70
LOC103992023	RING-H2 finger protein ATL2-like
LOC103989914	Hexose carrier protein HEX6
LOC103991268	Probable WRKY transcription factor 50
LOC104000052	Putative UDP-glucuronate:xylan α-glucuronosyltransferase 3

HEX. hexose; NAC: NAM, ATAF1/2, CUC2; PR: pathogenesis-related; UDP:uridine diphosphate.

**Table 3:** Gene ontology (GO) terms detected in genes differentially expressed between the resistant and susceptible banana (*Musa* spp. AAA) cultivars before and after infection with *Fusarium oxysporum* f. sp. *cubense* race 4.

GO ID	Term	Percentage of DEGs before Infection (%)	Percentage of DEGs after Infection (%)
GO:0015706	Nitrate transport	4.17	4.17
GO:0006073	Cellular glucan metabolic process	7.69	7.69
GO:0005975	Carbohydrate metabolic process	4.29	2.86
GO:0010030	Positive regulation of seed germination	33.33	33.33
GO:0080167	Response to karrikin	6.82	2.27
GO:0009611	Response to wounding	7.04	1.41
GO:0010167	Response to nitrate	3.57	3.57
GO:0016998	Cell wall macromolecule catabolic process	16.67	16.67
GO:0009408	Response to heat	1.82	3.64
GO:0044264	Cellular polysaccharide metabolic process	25	25
GO:0006508	Proteolysis	3.77	0.94
GO:0009407	Toxin catabolic process	5.26	5.26
GO:0050832	Defense response to fungus	5.13	5.13
GO:0032259	Methylation	1.92	5.76
GO:0010583	Response to cyclopentenone	10	10
GO:0009409	Response to cold	1.08	1.08
GO:0006032	Chitin catabolic process	16.67	16.67
GO:0009736	Cytokinin-activated signaling pathway	20	20
GO:0009505	Plant-type cell wall	5.56	4.17
GO:0005618	Cell wall	1.34	0.67
GO:0005887	Integral component of plasma membrane	11.11	11.11
GO:0043231	Intracellular membrane-bounded organelle	4.83	1.61
GO:0044444	Cytoplasmic part	3.13	3.13
GO:0009506	Plasmodesma	0.47	0.94
GO:0016020	Membrane	1.13	0.23
GO:0005773	Vacuole	1.33	1.33
GO:0003700	Sequence-specific DNA binding transcription factor activity	1.22	2.45
GO:0008168	Methyltransferase activity	2.5	7.5
GO:0016757	Transferase activity, transferring glycosyl groups	4.76	2.38
GO:0004568	Chitinase activity	12.5	12.5
GO:0004674	Protein serine/threonine kinase activity	0.63	0.63
GO:0000166	Nucleotide binding	0.61	0.61
GO:0004601	Peroxidase activity	4	4
GO:0030599	Pectinesterase activity	10	10
GO:0004722	Protein serine/threonine phosphatase activity	3.33	3.33
GO:0016762	Xyloglucan:xyloglucosyl transferase activity	7.69	7.69
GO:0004190	Aspartic-type endopeptidase activity	6.67	6.67

DEG: differentially expressed genes; GO: gene ontology; NAC: NAM, ATAF1/2, CUC2.

**Table 4:** GO items and relevant genes significantly accumulated in resistant and susceptible banana (*Musa* spp. AAA) cultivars after infection with *Fusarium oxysporum* f. sp. *cubense* race 4.

Comparison Pair	GO Annotation	Gene ID	Gene Description
YKCK–YKT	RNA splicing	LOC103989140	Uncharacterized
	Cellular zinc ion homeostasis	LOC103984038	α-carbonic anhydrase 7-like
	One-carbon metabolic process		
	Carbonate dehydratase activity		
	Calcium-transporting ATPase activity		
BXCK–BXT	Anaerobic respiration	LOC103970727	Kelch repeat-containing protein At3g27220-like
		LOC103984439	Uncharacterized
	Iron ion binding	LOC103985201	Cytochrome c-like
		LOC103996064	Glutamate synthase 1 [NADH], chloroplastic-like
		LOC103990398	Abscisic acid 8′-hydroxylase 1-like
		LOC103974663	Trans-cinnamate 4-monooxygenase-like
		LOC103972072	Cytochrome P450 84A1-like
	Heme binding	LOC103985201	Cytochrome c-like
		LOC103990398	Abscisic acid 8′-hydroxylase 1-like
		LOC103974663	Trans-cinnamate 4-monooxygenase-like
		LOC103972072	Cytochrome P450 84A1-like
	Electron carrier activity	LOC103985201	Cytochrome c-like
		LOC103990398	Abscisic acid 8′-hydroxylase 1-like
		LOC103974663	Trans-cinnamate 4-monooxygenase-like
		LOC103972072	Cytochrome P450 84A1-like
BXT–YKT	DNA binding	LOC103972990	Protein CUP-SHAPED COTYLEDON 3-like
		LOC103984324	Uncharacterized
		LOC103982987	Auxin-responsive protein IAA4-like
		LOC103985820	NAC domain-containing protein 21/22-like
		LOC103990974	TF JUNGBRUNNEN 1-like
		LOC103975993	Ethylene-responsive TF ERF107-like
		LOC103993650	Ethylene-responsive TF 11-like
		LOC103992490	NAC domain-containing protein 68-like
		LOC103971736	TF MYB6-like

BX: “Baxijiao”, highly susceptible; YK: “Yueyoukang 1”, highly resistant. CK: control; T: treatment with pathogen. GO: gene ontology; TF: transcription factor.

**Table 5:** KEGG pathways of genes differentially expressed between two banana (*Musa* spp. AAA) cultivars before and after infection with *Fusarium oxysporum* f. sp. *cubense* race 4.

KEGG ID	Term
mus00020	Citrate cycle
mus00100	Steroid biosynthesis
mus00260	Glycine, serine and threonine metabolism
mus00280	Valine, leucine and isoleucine degradation
mus00310	Lysine degradation
mus00450	Selenocompound metabolism
mus00710	Carbon fixation in photosynthetic organisms
mus00750	Vitamin B6 metabolism
mus03040	Spliceosome
mus04145	Phagosome

KEGG: Kyoto encyclopedia of genes and genomes.

**Table 6:** The top 21 Kyoto encyclopedia of genes and genomes (KEGG) pathways with corrected *p* value ≤ 0.05.

Comparison Pair	Pathway ID	Pathway	No. of DEGs	Up-Regulated DEGs	Down-Regulated DEGs
BXCK–YKCK	mus01110	Biosynthesis of secondary metabolites	52	36	16
	mus00940	Phenylpropanoid biosynthesis	19	13	6
	mus00941	Flavonoid biosynthesis	9	9	0
	mus00360	Phenylalanine metabolism	14	5	9
	mus01100	Metabolic pathways	68	39	29
	mus00960	Tropane, piperidine and pyridine alkaloid biosynthesis	5	4	1
	mus00950	Isoquinoline alkaloid biosynthesis	4	3	1
	mus00500	Starch and sucrose metabolism	12	4	8
	mus00480	Glutathione metabolism	6	5	1
	mus00100	Steroid biosynthesis	4	0	4
YKCK–YKT	mus04712	Circadian rhythm—plant	2	1	1
BXCK–BXT	mus01100	Metabolic pathways	44	13	31
	mus01110	Biosynthesis of secondary metabolites	27	11	16
	mus00430	Taurine and hypotaurine metabolism	4	0	4
	mus00940	Phenylpropanoid biosynthesis	9	5	4
	mus00010	Glycolysis/Gluconeogenesis	6	0	6
	mus00500	Starch and sucrose metabolism	7	0	7
	mus00360	Phenylalanine metabolism	5	3	2
	mus00941	Flavonoid biosynthesis	3	2	1
	mus00250	Alanine, aspartate and glutamate metabolism	3	0	3
	mus00592	α-Linolenic acid metabolism	3	1	2

BX: “Baxijiao”, a cultivar highly susceptible to *Fusarium oxysporum* f. sp. *cubense* race 4; YK: “Yueyoukang 1”, a cultivar highly resistant to *Fusarium oxysporum* f. sp. *cubense* race 4. CK: control; T: treatment with pathogen. DEG: differentially expressed genes; KEGG: Kyoto encyclopedia of genes and genomes.

## Supplementary Materials

Supplementary materials can be found at http://www.mdpi.com/1422-0067/19/2/350/s1.

## Abbreviations

BP	Biological process
BSA	Bovine serum albumin
CC	Cellular compartment
DEG	Differentially expressed genes
DGE	Digital gene expression
FDR	False discovery rate
*Foc*	*Fusarium oxysporum* f. sp. *cubense*
*Foc*4	Race 4 of *Foc*
GO	Gene ontology
KEGG	Kyoto encyclopedia of genes and genomes
LOB	Lateral organ boundaries
LRR	Leucine-rich repeat
MF	Molecular function
NAC	NAM, ATAF1/2, CUC2
PBS	Phosphate-buffered saline
PDA	Potato dextrose agar
PR	Pathogenesis-related
qRT-PCR	Quantitative real time PCR
RPKM	Reads per kilobase of transcript per million mapped reads
TFs	Transcription factors
UDP	Uridine diphosphate
