# Supplementary material for: Comparative Digital Gene Expression Analysis of Tissue-Cultured Plantlets of Highly Resistant and Susceptible Banana Cultivars in Response to Fusarium oxysporum
Source: Int J Mol Sci. 2018 Jan 24;19(2):350. doi: 10.3390/ijms19020350 (PMC5855572; doi:10.3390/ijms19020350)
Supplement: Supplementary file 1 [file ijms-19-00350-s001.zip › suppl. final/ijms-255688-supplementary/Figure S1-S12 final.pdf]

International Journal of
Molecular Sciences

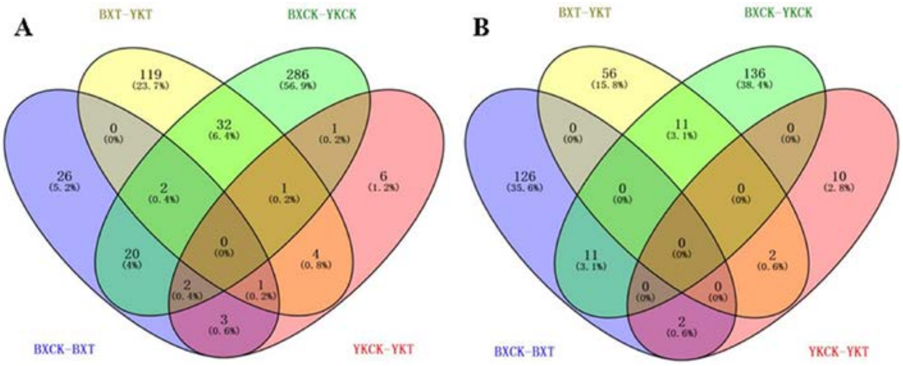

Figure S1. Venn diagram of genes differentially expressed in resistant and susceptible banana (*Musa* spp. AAA) cultivars before and after pathogen attack. (**A**) Up-regulated genes; (**B**) Down-regulated genes. BX: "Baxijiao", highly susceptible to *Fusarium oxysporum* f.sp *cubense* tropical race 4; YK: "Yueyoukang 1", highly resistant to *Fusarium oxysporum* f.sp *cubense* race 4. CK: the control; T: treatment with pathogen.

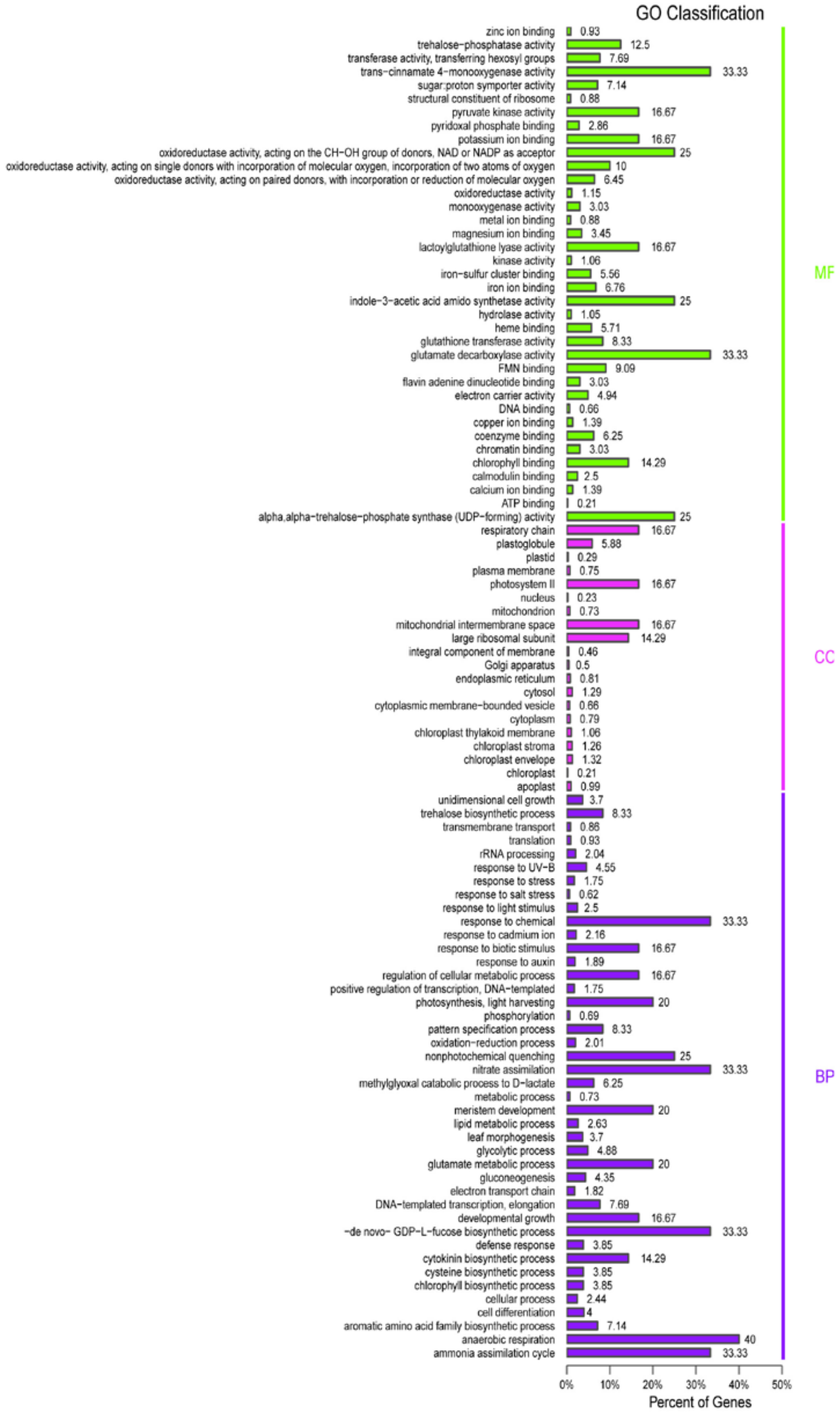

Figure S2. Gene ontology annotations enriched in the susceptible banana cultivar (*Musa* spp. AAA cv. Baxijiao) after infection with *Fusarium oxysporum* f. sp. *Cubense***.** BP: biological process; CC: cellular compartment; MF: molecular function.

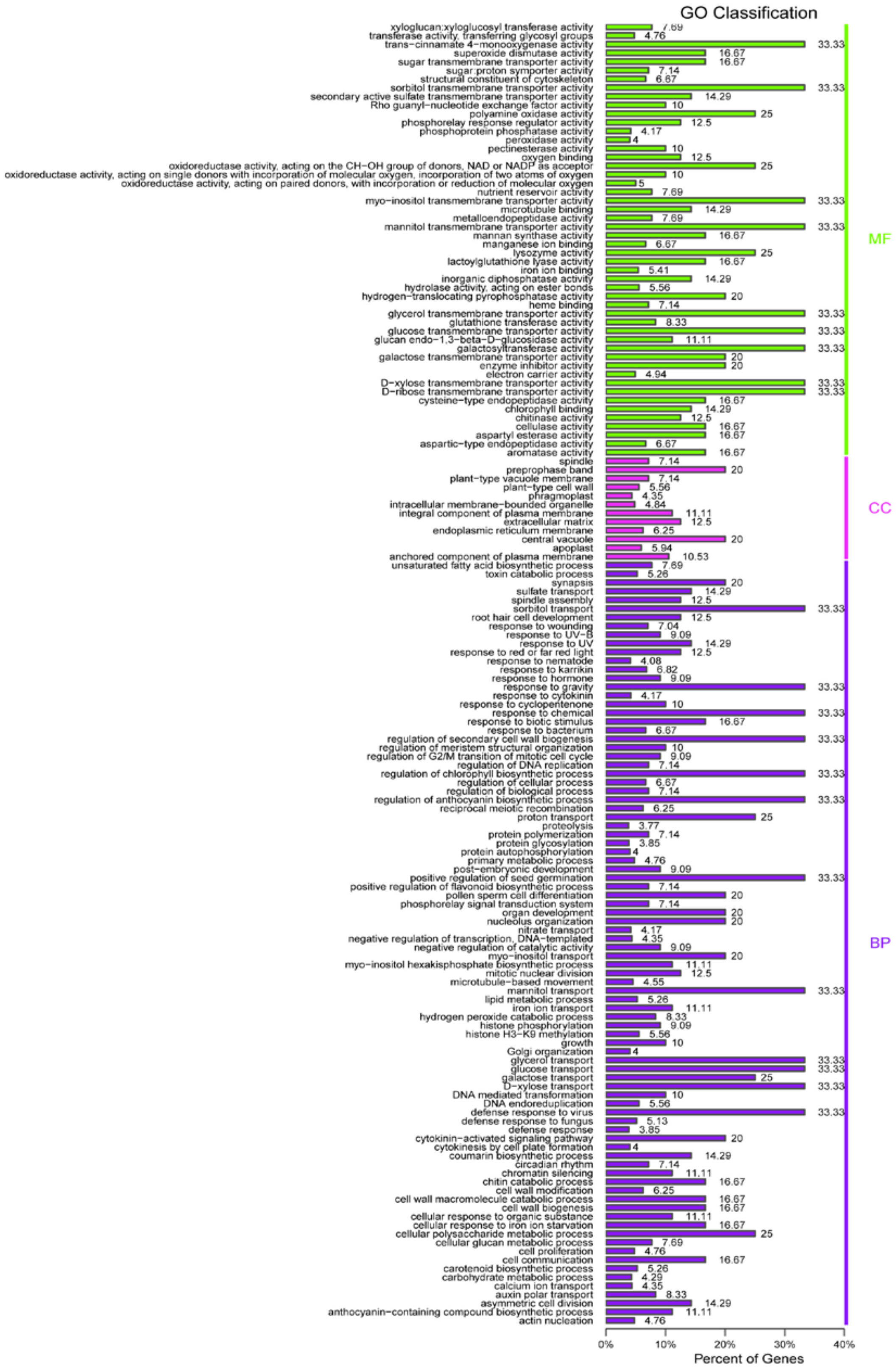

Figure S3. Gene ontology annotations present between the susceptible (*Musa* spp. AAA cv. Baxijiao) and resistant (*Musa* spp. AAA cv. Yueyoukang 1) banana cultivars. BP: biological process; CC: cellular compartment; MF: molecular function.

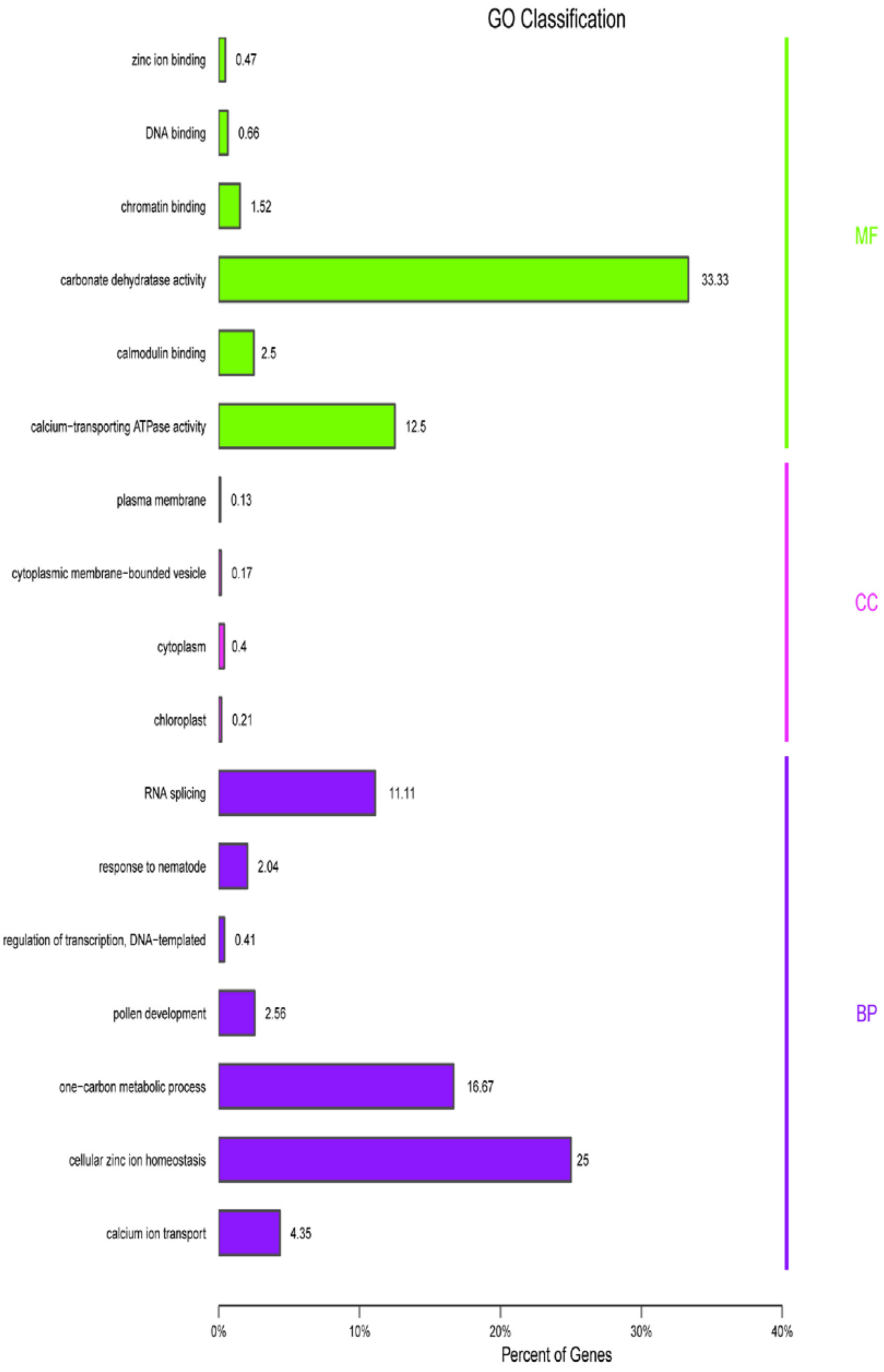

Figure S4. Gene ontology annotations enriched in the resistant banana cultivar (*Musa* spp. AAA cv. Yueyoukang 1) after infection with *Fusarium oxysporum* f. sp. *Cubense***.** BP: biological process; CC: cellular compartment; MF: molecular function.

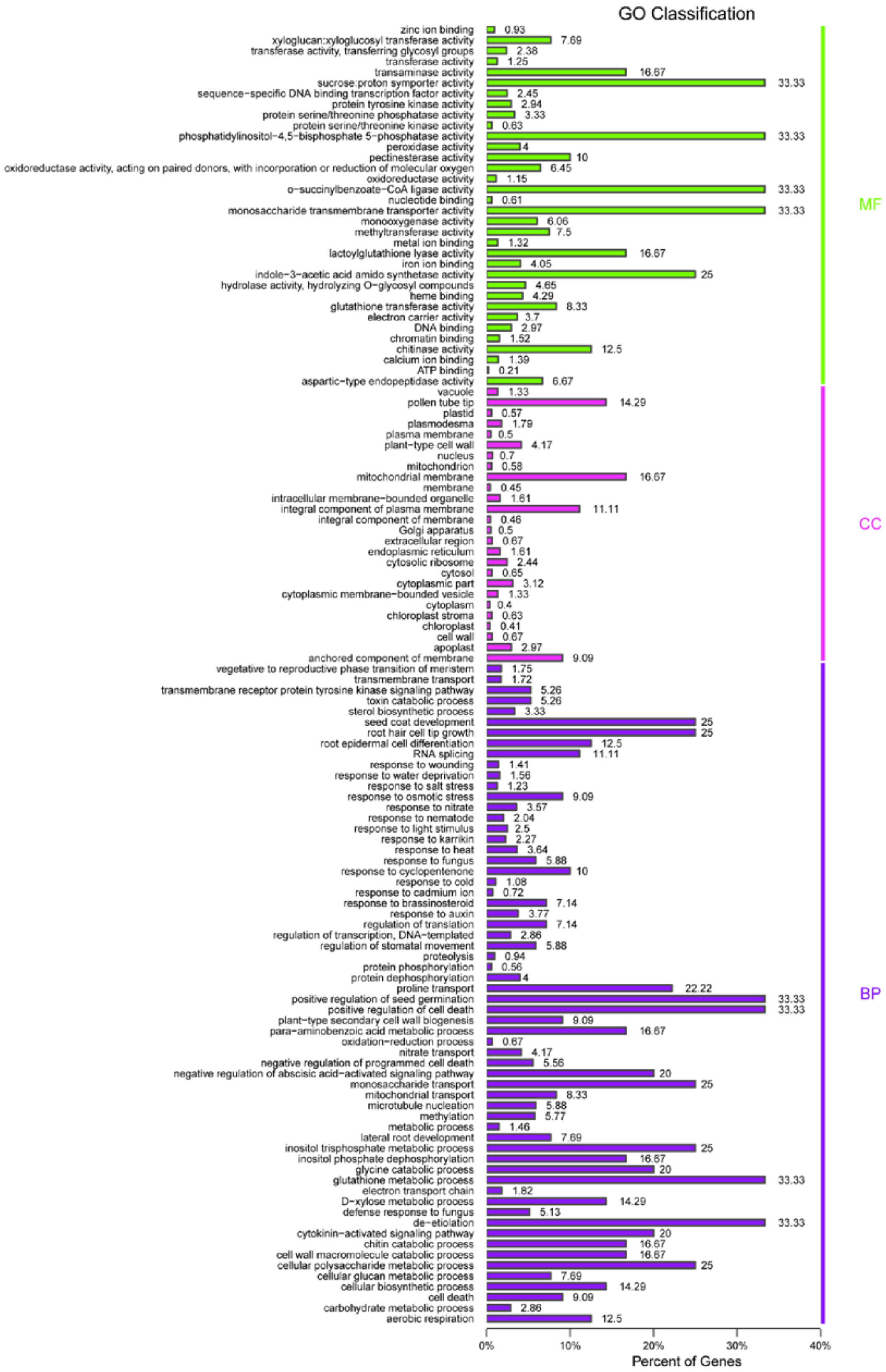

Figure S5. Gene ontology annotations present between the susceptible (*Musa* spp. AAA cv. Baxijiao) and resistant (*Musa* spp. AAA cv. Yueyoukang 1) banana cultivars after infection with *Fusarium oxysporum* f. sp. *Cubense.* BP: biological process; CC: cellular compartment; MF: molecular function.

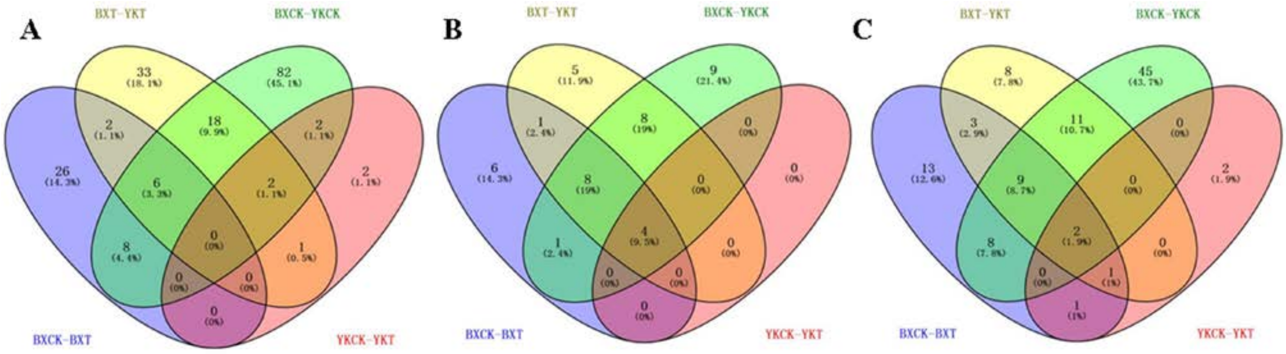

Figure S6. The Venn diagram of GO annotation items in resistant and susceptible banana (*Musa* spp. AAA) cultivars before and after pathogen attack. (**A**) Biological process; (**B**) Cellular compartment; **(C)** Molecular function. BX: "Baxijiao", highly susceptible to *Fusarium oxysporum* f.sp *cubense* race 4; YK: "Yueyoukang 1", highly resistant to *Fusarium oxysporum* f.sp *cubense* race 4. CK: the control; T: treatment with pathogen.

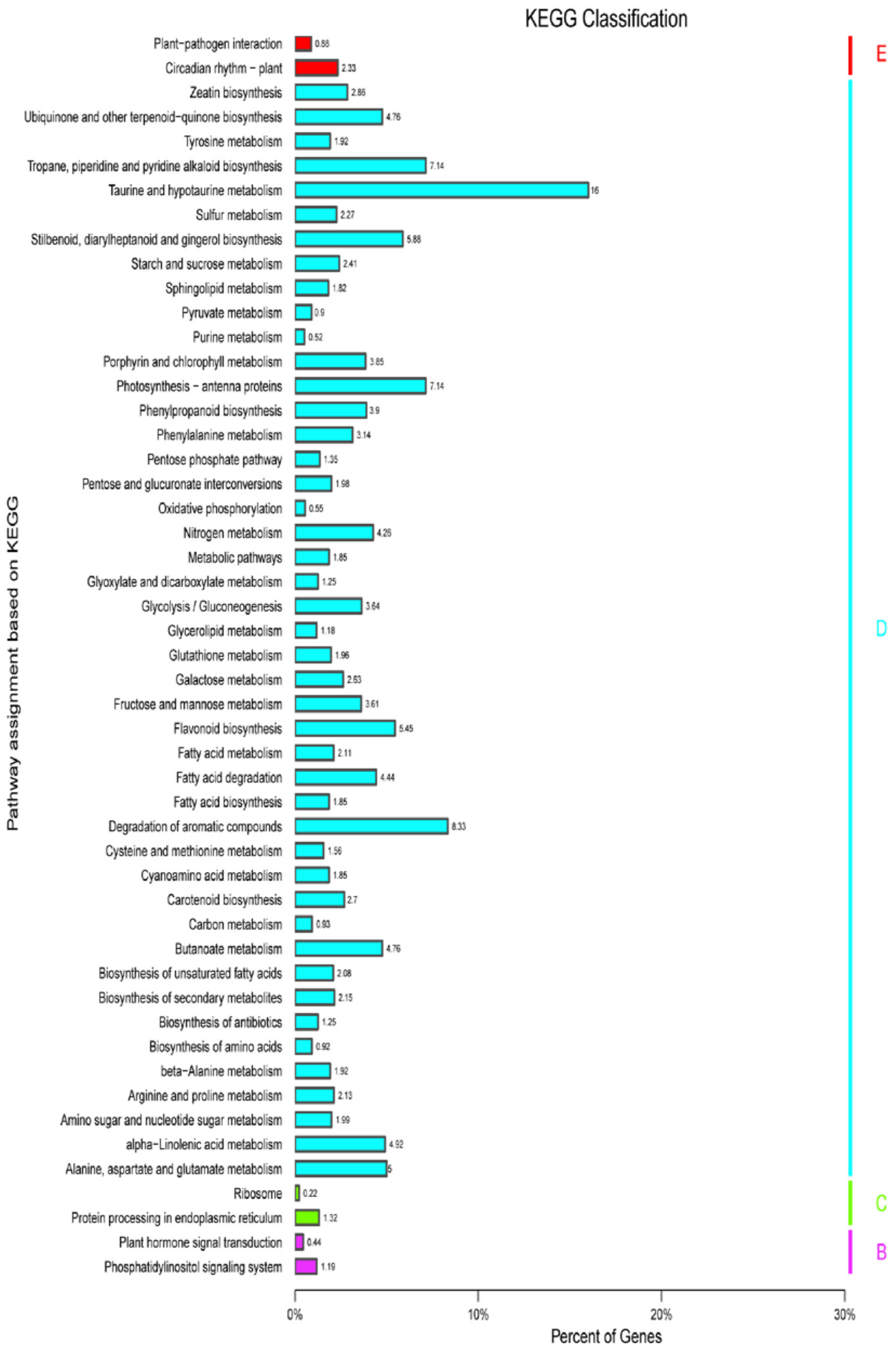

Figure S7. Kyoto encyclopedia of genes and genomes (KEGG) pathways enriched in the susceptible banana cultivar (*Musa* spp. AAA cv. Baxijiao) after infection with *Fusarium oxysporum* f. sp. *Cubense*. **B**: Environmental information processing; **C**: Genetic information processing; **D**: Metabolism; E: Organismal systems.

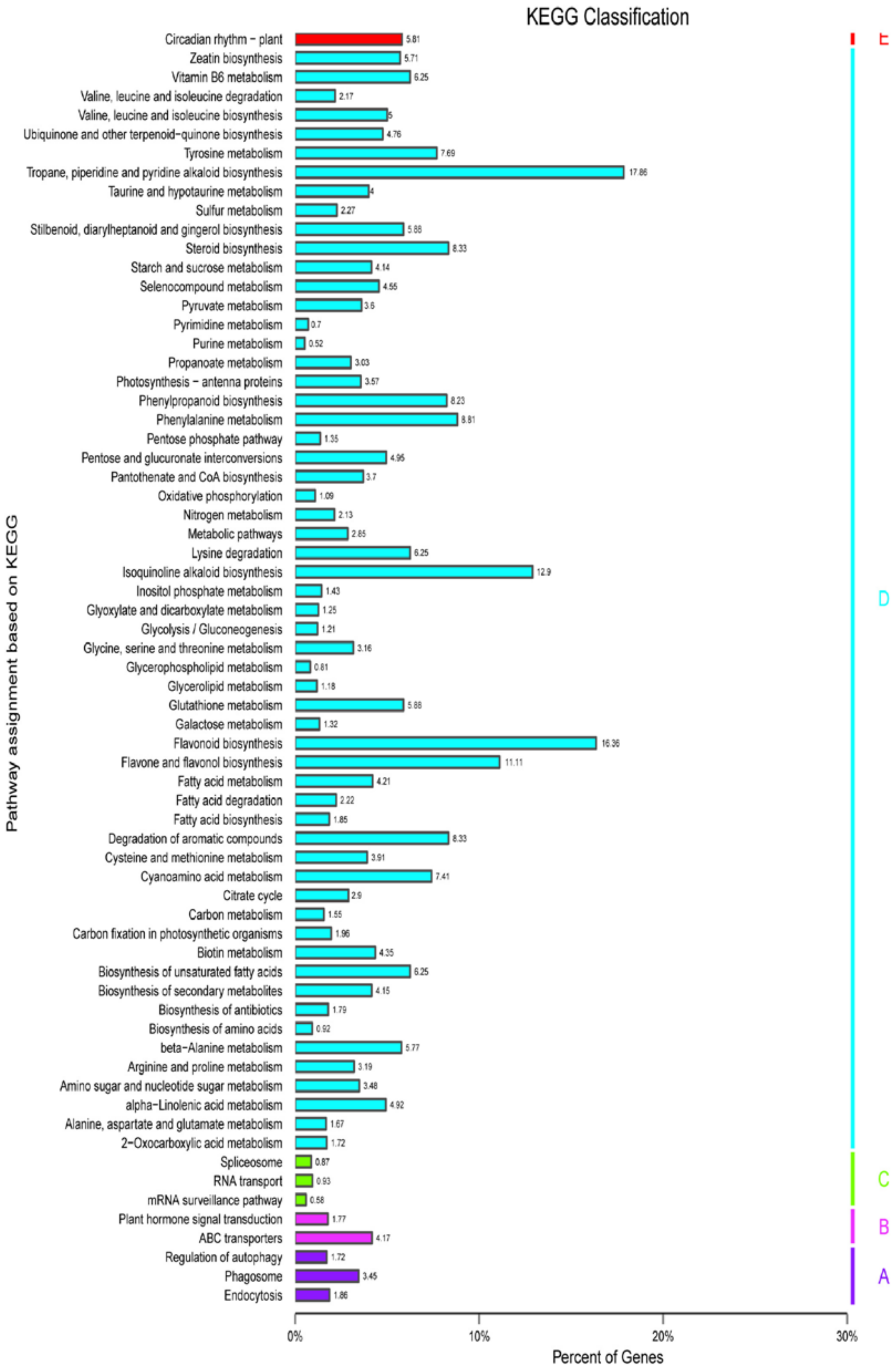

Figure S8. Kyoto encyclopedia of genes and genomes (KEGG) pathways present between the susceptible (*Musa* spp. AAA cv. Baxijiao) and resistant (*Musa* spp. AAA cv. Yueyoukang 1) banana cultivars. **A**: Cellular processes; **B**: Environmental information processing; **C**: Genetic information processing; D: Metabolism; E: Organismal systems.

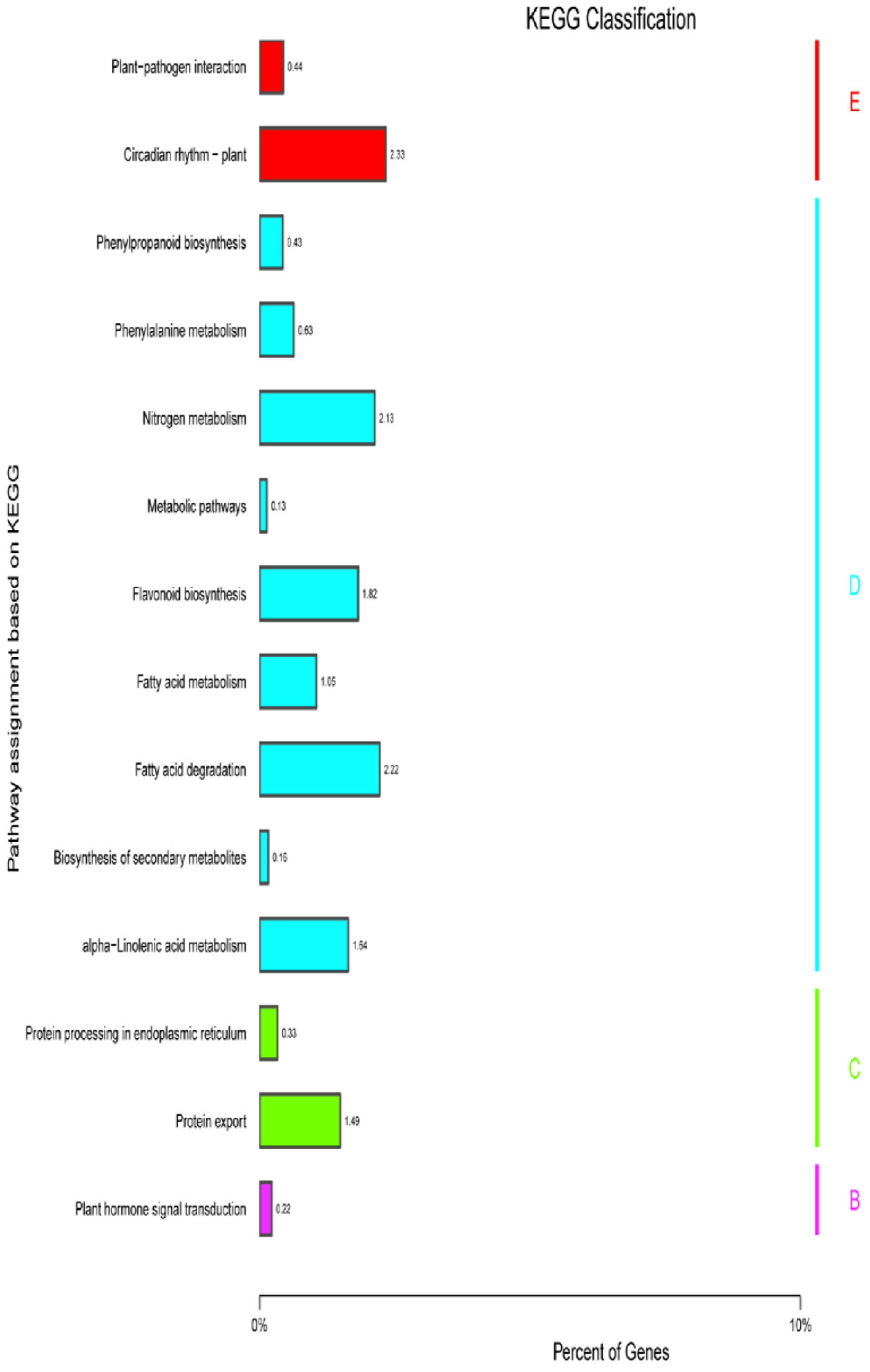

Figure S9. Kyoto encyclopedia of genes and genomes (KEGG) pathways enriched in the resistant banana cultivar (*Musa* spp. AAA cv. Yueyoukang 1) after infection with *Fusarium oxysporum* f. sp. *cubense*.. B: Environmental information processing; C: Genetic information processing; D: Metabolism; E: Organismal systems

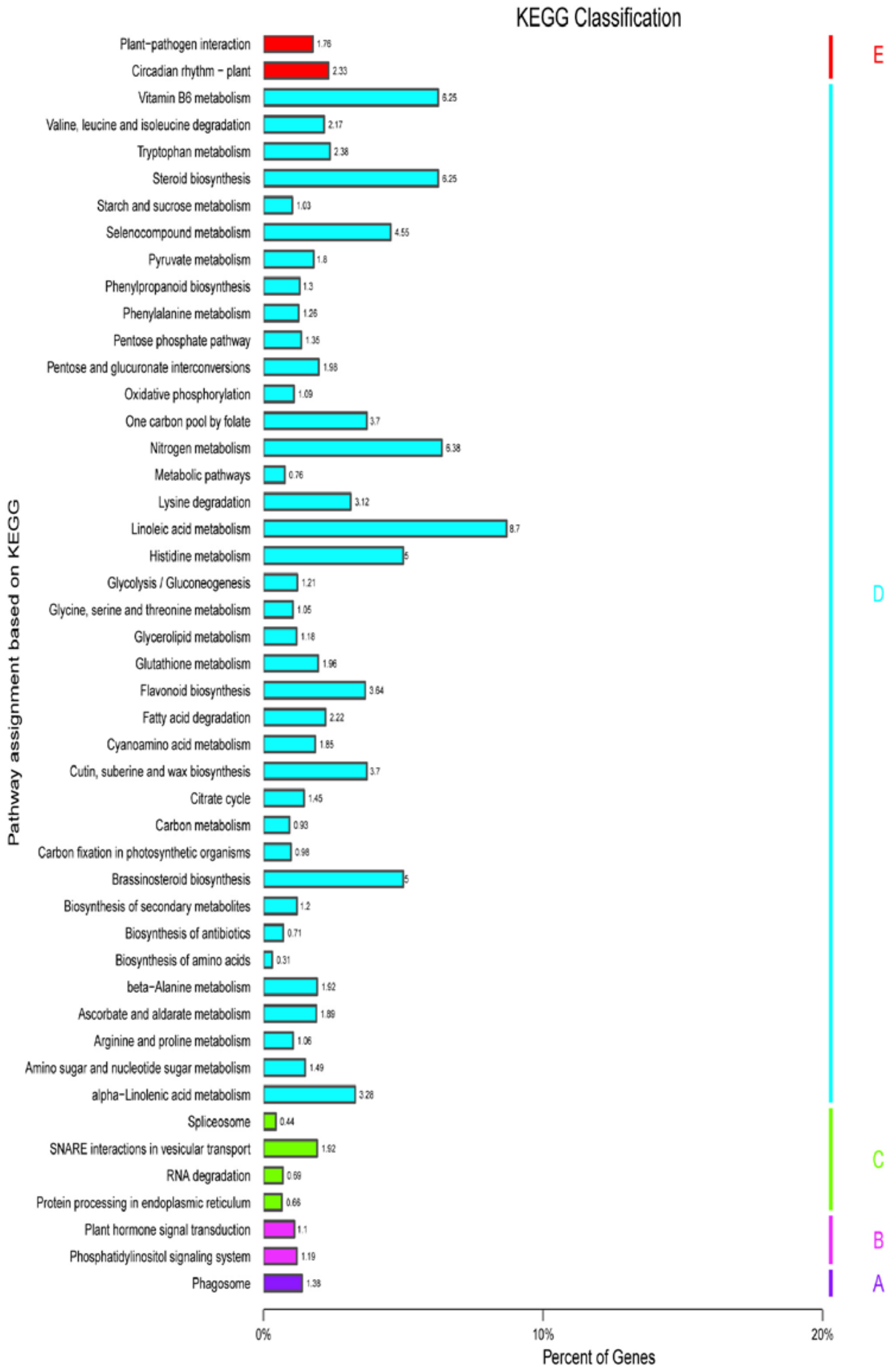

Figure S10. Kyoto encyclopedia of genes and genomes (KEGG) pathways present between the susceptible (*Musa* spp. AAA cv. Baxijiao) and resistant (*Musa* spp. AAA cv. Yueyoukang 1) banana cultivars after infection with *Fusarium oxysporum* f. sp. *cubense*. **A**: Cellular processes; **B**: Environmental information processing; **C**: Genetic information processing; **D**: Metabolism; **E**: Organismal systems

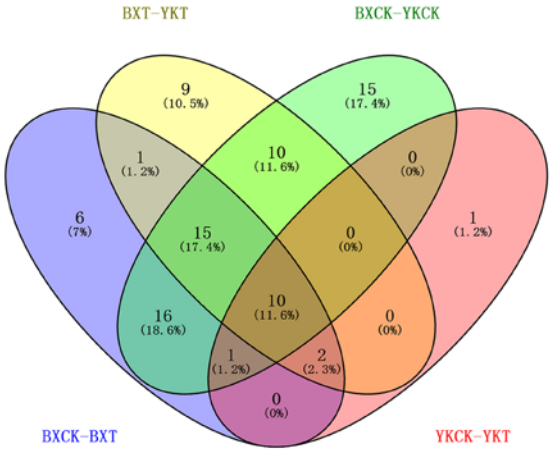

Figure S11. The Venn diagram of accumulated KEGG pathways in resistant and susceptible banana (*Musa* spp. AAA) cultivars before and after pathogen attack. BX: "Baxijiao", highly susceptible to *Fusarium oxysporum* f.sp *cubense* race 4; YK: "Yueyoukang 1", highly resistant to *Fusarium oxysporum* f.sp *cubense* race 4. CK: the control; T: treatment with pathogen.

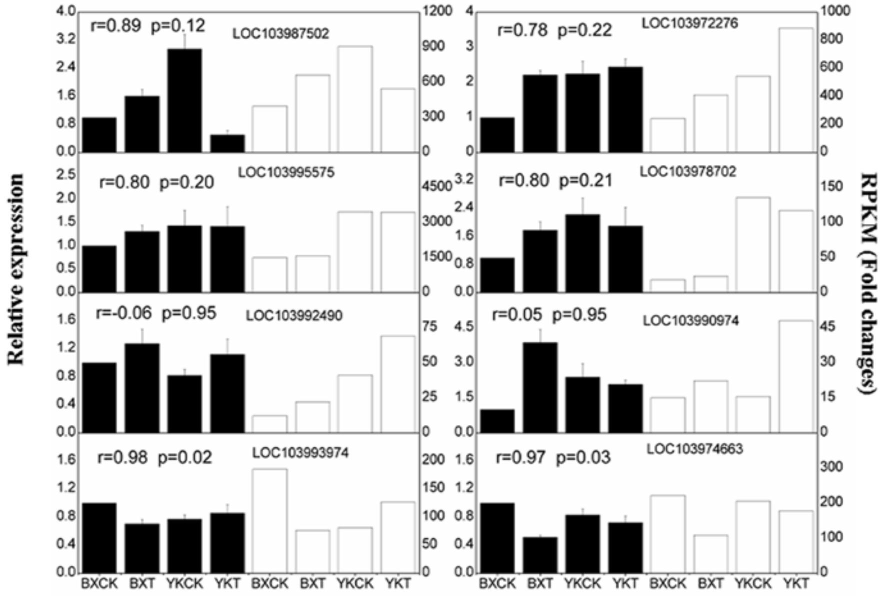

Figure S12. Validation of DGE data by *q*RT-PCR. Graphs show the relative expression of selected genes as examined by *q*RT-PCR (black columns), and their corresponding RPKM values from RNA-Seq (white columns). Error bars are standard errors. BX: "Baxijiao", highly susceptible to *Fusarium oxysporum* f.sp *cubense* race 4; YK: "Yueyoukang 1", highly resistant to *Fusarium oxysporum* f.sp *cubense* race 4. CK: the control; T: treatment with pathogen. DGE: digital gene expression; RPKM: reads per kilobase of transcript per million mapped reads.
