# Supplementary material for: Comparative Digital Gene Expression Analysis of Tissue-Cultured Plantlets of Highly Resistant and Susceptible Banana Cultivars in Response to Fusarium oxysporum
Source: Int J Mol Sci. 2018 Jan 24;19(2):350. doi: 10.3390/ijms19020350 (PMC5855572; doi:10.3390/ijms19020350)
Supplement: Supplementary file 1 [file ijms-19-00350-s001.zip › suppl. final/ijms-255688-supplementary/Table S1-2+7-10 final.pdf]

Table S1. Output statistics of the transcriptome of "Baxijiao" and "Yueyoukang 1" (*Musa* spp. AAA) inoculated with *Fusarium oxysporum* f. sp. *cubense* race 4.

Sample	Clean Reads	Total Mapped	Unique Mapped	Multiple Mapped	GC（%）	Q20（%）	Q30（%）
BXCK-1	14488133（98.41）	93.88	75.13	18.75	53	98.84	96.38
BXCK-2	12108282（97.95）	94.09	75.79	18.31	51	98.91	96.55
BXT-1	1002529（98.05%）	89.77	71.26	18.51	51	98.45	94.99
BXT-2	12846604（98.54）	92.20	66.37	25.83	52	98.91	96.49
YKCK-1	14536477（98.41）	93.98	72.50	21.48	52	98.85	96.4
YKCK-2	13405320（98.37）	94.29	69.05	25.24	53	98.84	96.37
YKT-1	11389816（98.03）	87.63	70.02	17.61	51	98.85	96.41
YKT-2	15457032（97.67）	87.59	64.63	22.96	53	98.84	96.33

BX: "Baxijiao", highly susceptible; YK: "Yueyoukang 1", highly resistant. CK: the control; T: treatment with the pathogen. Multiple mapped: the percentage of multiple mapped reads to total clean reads; Total mapped: the percentage of these clean reads that could be mapped to the reference database; Uniquely mapped: the percentage of unique mapped reads to total clean reads. Q20 means base quality more than 20; Q30 means base quality more than 30.

Table S2. Numbers of differentially expressed genes in two banana (*Musa* spp. AAA) cultivars before and after infection with *Fusarium oxysporum* f.sp *cubense* race 4.

Comparison Pair	No. of Differentially Expressed Genes	No. of Up-Regulated Genes	No. of Down-Regulated Genes
BXCK–YKCK	502	344	158
YKCK–YKT	32	18	14
BXCK–BXT	193	54	139
BXT–YKT	228	159	69
Total	955	575	380

BX: "Baxijiao", highly susceptible; YK: "Yueyoukang 1", highly resistant. CK: the control; T: treatment with pathogen.

Table S7. Numbers of GO items and KEGG pathways found in each comparison.

Comparison Pair	GO				KEGG
	Biological Process	Cellular Component	Molecular Function	Total	
BXCK–YKCK	118	30	75	223	67
YKCK–YKT	7	4	6	17	14
BXCK–BXT	42	20	37	99	51
BXT–YKT	62	26	34	122	47

BX: "Baxijiao", highly susceptible to *Fusarium oxysporum* f.sp *cubense* race 4; YK: "Yueyoukang 1", highly resistant to *Fusarium oxysporum* f.sp *cubense* race 4. CK: the control; T: treatment with pathogen. GO: gene ontology; KEGG: Kyoto encyclopedia of genes and genomes.

Table S8. GO terms detected exclusively in the susceptible banana (*Musa* spp. AAA) cultivar after infection with *Fusarium oxysporum* f.sp *cubense* race 4.

GO ID	Term	DEGs	Background Genes	Percentage of DEGs (%)
GO:0009073	Aromatic amino acid family biosynthetic process	1	14	7.14
GO:0019344	Cysteine biosynthetic process	1	26	3.85
GO:0009061	Anaerobic respiration	2	5	40
GO:0019676	Ammonia assimilation cycle	1	3	33.33
GO:0009765	Photosynthesis, light harvesting	1	5	20
GO:0006536	Glutamate metabolic process	1	5	20
GO:0010196	Nonphotochemical quenching	1	4	25
GO:0005992	Trehalose biosynthetic process	1	12	8.33
GO:0009965	Leaf morphogenesis	1	27	3.70
GO:0042351	De "novo"' GDP-L-fucose biosynthetic process	1	3	33.33
GO:0006412	Translation	1	108	0.93
GO:0030154	Cell differentiation	1	25	4
GO:0006096	Glycolytic process	2	41	4.88
GO:0009826	Unidimensional cell growth	1	27	3.70

GO:0006354	DNA-templated transcription, elongation	1	13	7.69
GO:0015995	Chlorophyll biosynthetic process	1	26	3.85
GO:0009691	Cytokinin biosynthetic process	1	7	14.29
GO:0031323	Regulation of cellular metabolic process	1	6	16.67
GO:0048589	Developmental growth	1	6	16.67
GO:0045893	Positive regulation of transcription, DNA-templated	1	57	1.75
GO:0006364	rRNA processing	1	49	2.04
GO:0007389	Pattern specification process	1	12	8.33
GO:0019243	Methylglyoxal catabolic process to D-lactate	1	16	6.25
GO:0048507	Meristem development	1	5	20
GO:0042128	Nitrate assimilation	1	3	33.33
GO:0006094	Gluconeogenesis	1	23	4.35
GO:0015934	Large ribosomal subunit	1	7	14.29
GO:0009941	Chloroplast envelope	2	152	1.32
GO:0070469	Respiratory chain	1	6	16.67
GO:0005758	Mitochondrial intermembrane space	1	6	16.67
GO:0009523	Photosystem II	1	6	16.67
GO:0010287	Plastoglobule	1	17	5.88
GO:0010181	FMN binding	1	11	9.09
GO:0004805	Trehalose-phosphatase activity	1	8	12.5
GO:0003735	Structural constituent of ribosome	1	113	0.88
GO:0000287	Magnesium ion binding	1	29	3.45
GO:0030170	Pyridoxal phosphate binding	1	35	2.86
GO:0050662	Coenzyme binding	1	16	6.25
GO:0030955	Potassium ion binding	1	6	16.67
GO:0003825	α,α-trehalose-phosphate synthase (UDP-forming) activity	1	4	25
GO:0004743	Pyruvate kinase activity	1	6	16.67
GO:0005507	Copper ion binding	1	72	1.39
GO:0016758	Transferase activity, transferring hexosyl groups	1	13	7.69
GO:0004351	Glutamate decarboxylase activity	1	3	33.33
GO:0051536	Iron-sulfur cluster binding	1	18	5.56

DEG: differentially expressed genes; GDP: guanosine diphosphate; GO: gene ontology; UDP: uridine diphosphate.

Table S9. The changes in digital gene expression levels of 113 genes included in the 21 KEGG pathways.

Gene ID	log_2FoldChange				Description
	YKCK -BXCK	YKCK-YKT	BXCK -BXT	YKT -BXT	
LOC103968460	−1.05				Copper amine oxidase 1-like
LOC103968789		1.59			Curcumin synthase 3-like
LOC103969029	1.31				α-aminoadipic semialdehyde Synthase
LOC103969416	1.79				Peroxidase P7-like
LOC103969483	3.08				Tyrosine/DOPA decarboxylase 2-like
LOC103969489	−1.26				Pyruvate, phosphate dikinase, chloroplastic-like
LOC103970201			−1.61		Oxygen-dependent coproporphyrinogen-III oxidase, chloroplastic-like
LOC103970367	1.16				Naringenin,2-oxoglutarate 3-dioxygenase-like
LOC103970573	1.87			1.11	β-glucosidase 32-like
LOC103970644	1.30				Glutathione S-transferase U10-like
LOC103971032	−1.09				Peroxidase P7-like
LOC103971627	1.34	1.01			Phenylalanine ammonia-lyase-like
LOC103971898	1.42				Caffeic acid 3-O-methyltransferase-like
LOC103971934			4.80		caffeic acid 3-O-methyltransferase-like
LOC103971966	1.14				Pectinesterase-like
LOC103972072			−1.06		Cytochrome P450 84A1-like
LOC103972232	−1.23				Peroxidase 4
LOC103972276	1.22				Tropinone reductase homolog At1g07440-like
LOC103972421			−1.18		Coproporphyrinogen-III oxidase 1, chloroplastic-like
LOC103972970	1.64				Probable serine acetyltransferase 4
LOC103973048	1.30				Inorganic pyrophosphatase 2-like
LOC103973269	−1.83			−1.73	Glutathione S-transferase T1-like
LOC103973278	−1.12				Probable prolyl 4-hydroxylase 3
LOC103973721			−1.28		Pyruvate decarboxylase 1-like
LOC103973892	−2.12		−1.71		α-dioxygenase 1-like
LOC103974015	−7.40			−5.00	Obtusifoliol 14-α demethylase-like
LOC103974254	−1.64				Inositol-3-phosphate synthase-like
LOC103974633			−3.46		Chlorophyll a-b binding protein CP24 10A, chloroplastic
LOC103975026	1.78				Chitinase 6-like
LOC103975191	−1.05				Pectinesterase-like
LOC103975240			Inf		Probable 4-coumarate--CoA ligase 2
LOC103975954			−1.64		Chlorophyll a-b binding protein of LHCII type 1-like
LOC103976154	1.65				Glutathione S-transferase U18-like
LOC103976711	−1.23		−1.30		Probable trehalose-phosphate phosphatase 6
LOC103976912	1.29				S-adenosylmethionine decarboxylase proenzyme-like
LOC103977198			3.59		Sphinganine C(4)-monooxygenase 1-like

LOC103977235	1.76			1.09	Phenylpropanoylacetyl-CoA synthase-like
LOC103977427	1.62				Pectinesterase-like
LOC103977461			–1.29		Probable α, α-trehalose-phosphate synthase [UDP-forming] 9
LOC103977576	–1.41		–1.49		Cytokinin hydroxylase-like
LOC103977807			–1.08		Pyruvate decarboxylase 1
LOC103978140	1.20				Phenylalanine ammonia-lyase-like
LOC103978344	1.79				Branched-chain-amino-acid aminotransferase 3, chloroplastic-like
LOC103978458			–1.01		Hexokinase-2-like
LOC103978680			–1.65		Pyruvate kinase, cytosolic isozyme-like
LOC103978702	2.96			–0.20	Chitinase 1-like
LOC103978704	2.30			1.90	Chitinase 1-like
LOC103978957	1.05	1.23	1.30		Glyoxysomal fatty acid β-oxidation multifunctional protein MFP-a
LOC103979831	1.16				Flavonoid 3'-monooxygenase
LOC103980720	3.49				Probable 4-coumarate--CoA ligase 3
LOC103981301	1.14				Probable monogalactosyldiacylglycerol synthase 3, chloroplastic
LOC103982813	1.54		1.32		Dihydroflavonol-4-reductase-like
LOC103982815	2.19				Caffeic acid 3-O-methyltransferase-like
LOC103983737	–1.30				V-type proton ATPase subunit G1-like
LOC103983874			1.06		Phenylalanine ammonia-lyase
LOC103984267	–1.19				Alanine-glyoxylate aminotransferase 2 homolog 3, mitochondrial-like
LOC103984290	–1.10				Lysosomal β-glucosidase-like
LOC103984640	–1.02				Probable rhamnose biosynthetic enzyme 1
LOC103984681	–1.03				Endoglucanase 10-like
LOC103984705			–1.42		1-aminocyclopropane-1-carboxylate oxidase 1-like
LOC103984843	–1.11				δ(24)-sterol reductase-like
LOC103985238	1.82				Chitinase 6-like
LOC103985435	–1.46				Malate dehydrogenase, chloroplastic-like
LOC103985734	–1.30			–1.20	Methylsterol monooxygenase 1-1-like
LOC103985805	–1.14				Methylsterol monooxygenase 1-1-like
LOC103986053	2.345		3.84		Curcumin synthase 2-like
LOC103986187			–3.22		Pyruvate decarboxylase 1-like
LOC103986257			–1.19		Pectinesterase-like
LOC103987320	1.65				Peroxidase 4-like
LOC103987502	1.27				Chalcone synthase 2-like
LOC103987608	1.18				dCTP pyrophosphatase 1-like
LOC103987658	–1.56				Chlorophyll a-b binding protein CP26, chloroplastic-like
LOC103987820	1.26				Peroxidase P7-like
LOC103987972	1.23				Chalcone synthase 2
LOC103988116	–1.92				Polygalacturonase-like
LOC103988144			–1.25		Alcohol dehydrogenase 3-like
LOC103988682	2.99		1.34	1.56	Chitinase 6

LOC103988860	1.26				Short-chain type dehydrogenase/reductase-like
LOC103989311	1.43		1.51		Glucose-6-phosphate 1-dehydrogenase, cytoplasmic isoform-like
LOC103989812	–2.50				Putative pectinesterase 11
LOC103989858			–3.75		2-aminoethanethiol dioxygenase-like
LOC103990171	1.63		2.47		Tropinone reductase homolog
LOC103990441	–1.83				L-lactate dehydrogenase A-like
LOC103990854			–1.24		Glutamine synthetase nodule isozyme-like
LOC103991241	–1.44				Peroxidase P7-like
LOC103992160	1.23				Trans-cinnamate 4-monooxygenase-like
LOC103992527	2.12				Peroxidase 4-like
LOC103993486			–1.16		UDP-glucuronate 4-epimerase 6-like
LOC103993974	–1.12		–1.26		Probable 2-aminoethanethiol dioxygenase
LOC103994393	–1.22				Pyruvate kinase, cytosolic isozyme-like
LOC103994655	1.73				Primary amine oxidase-like
LOC103995095			–1.56		Probable 2-aminoethanethiol dioxygenase
LOC103995522	1.18				Polyphenol oxidase, chloroplastic-like
LOC103996064			–1.38		Glutamate synthase 1 (NADH), chloroplastic-like
LOC103996072	1.13				Acidic endochitinase SE2-like
LOC103996857	1.15		1.19		Tropinone reductase homolog
LOC103996973	–1.06				ATP-citrate synthase β-chain protein 1
LOC103997199	–1.04		–1.02		β-glucosidase 13-like
LOC103997659	1.06				Glutathione S-transferase U17-like
LOC103997680	–1.02				Peroxidase 1-like
LOC103998074	2.00				β-glucosidase 22-like
LOC103998799			3.25		Lignin-forming anionic peroxidase-like
LOC103999070	2.38		2.27		Probable glutathione S-transferase parA
LOC103999483			–1.35	1.56	Peroxidase 24-like
LOC103999830	–1.04		–1.58		Probable trehalose-phosphate phosphatase 6
LOC104000178		–1.56	–1.29		Protein LHY-like
LOC104000773	1.06				Chalcone synthase 2-like
LOC103973932	2.80		4.86		Caffeic acid 3-O-methyltransferase-like
LOC103985201			–3.22		Cytochrome c-like
LOC103986947			–3.53		Probable prolyl 4-hydroxylase 7
LOC103994603			–1.24		1-Aminocyclopropane-1-carboxylate oxidase 1-like
LOC103995822			–2.00		Glutamate decarboxylase 4-like
LOC104000442			–1.04		GDP-mannose 4,6 dehydratase 1-like

BX: "Baxijiao", a cultivar highly susceptible to *Fusarium oxysporum* f.sp *cubense* race 4; YK: "Yueyoukang 1", a cultivar highly resistant to *Fusarium oxysporum* f.sp *cubense* race 4. CK: the control; T: treatment with pathogen. KEGG: Kyoto encyclopedia of genes and genomes; GDP: guanosine diphosphate; UDP: uridine diphosphate.

Table S10. Primers for qPCR.

Gene	Primer Sequences (Forward/ Reverse)	Production size (bp)
18S rRNA	CCTGAGAAACGGCTACCACAT	171

	CACCAGACTTGCCCTCCA	
LOC103978702	TACGGAGACAACTTGGACTGC<br>ACTCACGGAGTCGCTATCTCA	116
LOC103972276	AGCTGTGGTGGCTTTCTTGT<br>GGTTTAGAGGTTGCCGTTGA	100
LOC103987502	CACAGTATGCCAATCGTTGC<br>ATCGGACACGCTTAAACAGC	113
LOC103993974	GACCAAAATGGAGAGGAGGA<br>CTGTTGTCAGCCCTCGATGA	120
LOC103974663	CTGGTCAAGGACTTCGAGTTG<br>CTTCATCTCCCTCTCATGGTG	142
LOC103990974	GAGTCCAGGCATGGATGATT<br>AGGGAAGTTTGGTAGCAGGAC	132
LOC103992490	TGCACCTCCAATGCATAGAC<br>TCTGCCATCCTCCTATGCTAC	113
LOC103995575	GCCAGTACGGATGATGAAGTC<br>TGACAAGCACTACGACACTGG	135
